# Population Genomics of *Megalobrama* Provides Insights into Evolutionary History and Dietary Adaptation

**DOI:** 10.3390/biology11020186

**Published:** 2022-01-25

**Authors:** Jing Chen, Han Liu, Ravi Gooneratne, Yao Wang, Weimin Wang

**Affiliations:** 1 Key Lab of Agricultural Animal Genetics, Breeding and Reproduction of Ministry of Education, College of Fisheries, Huazhong Agricultural University, Wuhan 430070, China; cjing24511@163.com (J.C.); othnielartorious@gmail.com (Y.W.); 2 Key Lab of Freshwater Animal Breeding, Ministry of Agriculture and Rural Affairs, College of Fisheries, Huazhong Agricultural University, Wuhan 430070, China; 3 Faculty of Agriculture and Life Sciences, Lincoln University, Lincoln 7647, New Zealand; ravi.gooneratne@lincoln.ac.nz

**Keywords:** genome resequencing, *Megalobrama*, population structure, demographic history, feeding habits

## Abstract

**Simple Summary:**

*Megalobrama* is the economically most important freshwater fish genus in China. In recent years, germplasm resources of *Megalobrama* have been depleting as a result of environmental degradation and artificial factors. In this study, we established the whole genome database of *Megalobrama* populations using the whole genome re-sequencing technology, explored population genetic structure, and inferred comprehensive evolutionary relationships using principal component analysis and population structure analysis. Furthermore, employing selective sweep analysis, we identified candidate genes related to variations in feeding habits, revealing the molecular mechanisms of environmental adaptability in *Megalobrama* populations. Taken together, this study describes the population history and genetic diversity of *Megalobrama* populations and also the molecular mechanisms likely involved their environmental adaptability. These findings will make a substantial contribution to conservation and utilization of *Megalobrama* germplasm resources.

**Abstract:**

*Megalobrama*, a genus of cyprinid fish, is an economically important freshwater fish widely distributed in major waters of China. Here, we report the genome resequencing of 180 *Megalobrama* fish including *M.* *amblycephala*, *M. skolkovii*, *M. hoffmanni*, and *M. pellegrini*. Population structure indicated that geographically divergent *Megalobrama* populations were separated into six subgroups. A phylogenetic tree showed that *M. skolkovii* was more closely related to *M. pellegrini* than other species and *M. hoffmanni* was clustered apart from other *Megalobrama* species, showing a high nucleotide diversity in geographic groups. Treemix validated gene flow from *M. amblycephala* to *M. skolkovii*, suggesting that introgression may provide an important source of genetic variation in the *M. skolkovii* populations. According to the demographic history analysis, it is speculated that *Megalobrama* might have been originally distributed in the Pearl River with some spread to Hainan Island and northern China due to lower sea levels during the glacial period. Whole-genome selective sweeps analysis demonstrated that *M. amblycephala* likely developed an enhanced energy metabolism mostly through fatty acid degradation pathways whereas *M. hoffmanni* possibly regulate lipid absorption via the cholesterol metabolism pathway. Taken together, this study provides a valuable genomic resource for future genetic investigations aiming to improve genome-assisted breeding of *Megalobrama* species.

## 1. Introduction

*Megalobrama* is a cyprinid fish genus that belongs to the subfamily Cultrinae (Cypriniformes, Cyprinidae). It is one of the economically most important fish species, as well as the main aquaculture species in China. The genus *Megalobrama* contains four recognized species, *M. amblycephala*, *M. skolkovii*, *M. hoffmanni*, and *M. pellegrini* [1]. Previous studies categorized the four species mainly based on morphologic traits and geographic distribution. For instance, *M*. *amblycephala* is distinguished by the morphology of the upper orbital bone and oral fissure, and also the caudal peduncle depth/length ratio [2]. It is mainly distributed in large- and medium-sized lakes in the middle and lower branches of the Yangtze River [3]. *M. skolkovii* differs from the other three species in the longer length of the first swim bladder [2]. It inhabits the Amur, Yangtze, Yellow, and Minjiang rivers [4]. *M. hoffmanni* has pigment deposits on the scale base that form dark spots on its body surface and exists mostly in the Pearl River and Hainan Island [5]. *M*. *pellegrini* dwells in the middle and upper reaches of the Yangtze River and has a unique upper jaw and upper orbital bone shape [6]. Although previous studies have reported biological differences between *Megalobrama* species, the phylogenetic relationships among *M. amblycephala*, *M. skolkovii*, *M. hoffmanni*, and *M. pellegrini* remain unknown [7,8].

Four *Megalobrama* species have distinct feeding behaviors. For instance, *M. hoffmanni* is an omnivorous fish that feeds mostly on benthic creatures such as freshwater shellfish, river clams, organic detritus, and some aquatic plants [9]. *M. skolkovii* and *M. pellegrini* are omnivorous as well, however, they primarily feed on aquatic plants [10]. Importantly, in *M. pellegrini*, the development of the upper and lower jaws as well as the thickening of the stratum corneum have strengthened the scraping function, making it more conducive to feeding on stationary organisms [6]. *M. amblycephala* lives in aquatic plant-rich lakes and its diet consists primarily of aquatic plants, emergent plants, floating-leaf plants, and submerged plants. It is classified as herbivorous fish that mainly feeds on aquatic vascular plants [11]. Obviously, *Megalobrama* species must have evolved metabolic mechanisms that allow them to adapt to varying dietary compositions. However, it is not clear how *Megalobrama* species regulate carbohydrate, lipid, and protein metabolism to maintain energy balance in the body.

*Megalobrama* germplasm resources have been rapidly depleting in recent years as a result of many factors, including environmental degradation factors. There are a few reports on *Megalobrama* genetic resource conservation and population genetics [1,2,4]. By using population genomics, the genetic information of species diversity can be established with the advent of high-throughput sequencing [12,13]. For instance, whole genome re-sequencing has been used to investigate the phylogenetic relationships among differentiated species [14,15]. Using the whole genome of *M. amblycephala* as reference genome, this study was designed with the following objectives: (1) establish the whole genome database of *Megalobrama* population using the whole genome re-sequencing technology; (2) explore the genetic structure of populations and infer detailed evolutionary relationships by phylogenetic tree, principal component analysis (PCA), and admixture analysis; (3) illustrate the demographic history of *Megalobrama* species by pairwise sequentially Markovian coalescent (PSMC) method; (4) determine candidate genes related to variations in feeding habits utilizing selective sweeps analysis that would indicate the molecular mechanism (s) of environmental adaptability of *Megalobrama* populations. This study will ascertain the genetic diversity, population history, and improve the understanding of the evolution of *Megalobrama* populations and their molecular mechanisms of environmental adaptability. These findings will represent a significant contribution to conservation and utilization of *Megalobrama*’s germplasm resources.

## 2. Materials and Methods

### 2.1. Samples Information

A total of 180 samples, eight populations of *M. amblycephala* from Liangzi Lake (LZL; *n* = 12), Poyang Lake (PYL; *n* = 12), Yuni Lake (YNL; *n* = 12), Tiane Island (TEL; *n* = 12), Dongting Lake (DTL; *n* = 11), Jinsha River Reservoir of Hongan County (JS; *n* = 1), Shaoguan (SG; *n* = 2), Yingde (YD; *n* = 7); six populations of *M. skolkovii* from Fuyuan (FY; *n* = 12), JS (*n* = 12), Qiantang River (QT; *n* = 12), SG (*n* = 10) and YD (*n* = 5), DTL (*n* = 1); four populations of *M. hoffmanni* from Qingyuan (QY; *n* = 12), Zhaoqing (ZQ; *n* = 12), Fengkai (FK; *n* = 12), and Hainan (HN; *n* = 12), and one population of *M. pellegrini* from Longxi River (LX; *n* = 12) were collected. The fin tissues from all samples were preserved in 95% ethanol and stored at −80 °C.

### 2.2. Genome Sequencing and Sequence Alignment

Genomic DNA was extracted from the fin of each fish using a DNA extraction kit (Tiangen, Beijing, China). Paired-end sequencing libraries with an insert size of approximately 300–400 bp were constructed for each individual using the BGISEQ 500 platform and sequenced on an MGISEQ-2000 sequencer. After removing joint pollution and low-quality reads, the filtered high-quality clean data were for subsequent comparative analysis. The filtering was performed using SOAPnuke (v1.5.6) (http://github.com/BGI-flexlab/SOAPnuke (accessed on 9 September 2020)) software. The specific filtering conditions were as follows: removed reads containing adapter, low-quality reads (base quality value less than or equal to 15 bases account for 10%), and reads with N bases greater than 10%. All cleaned reads were mapped to the *M. amblycephala* reference genome (mixed assembly of second and third generations) using BWA-MEM (0.7.13-r1126) with default parameters [16]. The effective depth was approximately 16.98 × per individual (Table S1). The Samtools v1.9 software was used to convert the SAM format to BAM format [17]. The SortSam.jar tool of Picard v1.117 software was used to compare and sort the files.

### 2.3. Discovery of Genomic Variations

The variation detection was performed with the Genome Analysis Toolkit (GATK, version v3.3.0) with the UnifiedGenotyper method [18]. Single nucleotide polymorphisms (SNPs) were filtered both per population and per individual using GATK hard filtering [19] with the following stringent filtering criteria: QD < 2.0 || FS > 60.0 || MQ < 40.0 || ReadPosRankSum < −8.0 || MQRankSum < −12.5. Insertions and deletions (Indels) were filtered with parameters: QD < 2.0 || FS > 200.0 || ReadPosRankSum < −20.0. SNPs were pruned for minor allele frequency (MAF) >1% in each population. The snpEff (v4.3T) software was used to annotate SNPs and count the annotation information [20].

### 2.4. Population Structure Analysis and Species Definition Analysis

The principal component analysis (PCA) was performed using EIGENSOFT (version 4.2) and the significance level of eigenvectors was determined with the Tracy-Widom test [21]. Admixture version 1.3.0 was used to analyze the population structure [22]. 50 different random seeds were selected and indifferently analyzed *K*, and CLUMPP (v1.1.2) software was used to merge 50 analytical results [23]. DISTRUCT (v1.1) software was used to draw the population structure diagram [24]. Population structure was inferred by admixture with 10-fold cross-validation. Admixture was run for each possible group number (*K* = 2 to 8) with 200 bootstrap replicates. No admixed individuals were used to construct the phylogenetic tree by FastTree v2.1 software [25].

### 2.5. Linkage Disequilibrium and Diversity Analysis

Linkage disequilibrium (LD) is the non-random association between alleles of different loci in the population and *r*^2^ can be used to calculate the degree of linkage disequilibrium between two markers. In this study, the software PopLDdecay (v3.40) (https://github.com/BGI-shenzhen/PopLDdecay (accessed on 4 December 2020)) was used to set the parameters (-MaxDist 1000 -MAF 0.05 -Miss 0.1) to perform linkage disequilibrium analysis on SNPs [26]. LD is mostly affected by recombination, artificial selection, and population type. Generally, the distance at which each population decays to half of the maximum r^2^ value is used as the decay distance of the population. Based on all credible SNP data, vcftools (v0.1.13) software was used to calculate the nucleotides polymorphisms (π) of the four *Megalobrama* species and the differentiation index (Fst) between species [27].

### 2.6. Gene Flow Analysis

Treemix software was used to investigate the gene flow between populations [28]. The analysis utilized allele frequencies to construct a maximum likelihood tree and infer the migration events between populations. It is assumed that there are *n* migration events, and each migration event is calculated separately. Two analyses were performed. In one, the different *Megalobrama* populations were divided into four subgroups: *M. hoffmanni*, *M. skolkovii*, *M. amblycephala*, and *M. pellegrini*, with *M. hoffmanni* as the out group. In the other, *M. hoffmanni* was recognized as an outgroup with nine subgroups including *M. pellegrini* from Longxi River; *M. skolkovii* from Fuyuan, Shaoguan, Qiantang River, and Jinsha River Reservoir; and *M. amblycephala* from Dongting Lake, Poyang Lake, and Liangzi Lake added for gene flow analysis. 

### 2.7. Reconstruction of the Ancestral Geographic Distribution of Megalobrama

Based on the phylogenetic tree generated by the credible SNPs, we used the statistical dispersal–vicariance analysis (S-DIVA), dispersal-extinction-cladogenesis (DEC), and Bayesian binary MCMC (BBM) models of reconstruct ancestral state in phylogenies (RASP) 4.0 software to reconstruct the geographical distribution of *Megalobrama* ancestors [29,30]. In DEC, the possibility of gene exchange between non-adjacent distribution areas was limited to zero. In BBM, the MCMC chain was run for 10 million generations. The sampling frequency was 1000 and the first 25% samples were discarded as burn-in. The number of the most allowed distribution locations was set to 4. In the final analysis, the outgroup information was deleted. The division of the distribution area was mainly based on the Chinese water systems.

### 2.8. Demographic History Reconstruction and Estimates of the Divergence Time

The pairwise sequentially Markovian coalescent (PSMC) method was used to model the history of four *Megalobrama* species (at least 20 ×) and infer the historical changes in their effective population sizes and population separations. The PSMC model was utilized to estimate changes in the effective population size using heterozygous sites across the genome [31]. The number of free atomic time intervals (-p option) was reset with the initial value of r = θ/ρ (-r option) according to previous research [31]. Based on the estimates from the *M. amblycephala* genome, an average mutation rate (μ) of 0.1 × 10^−8^ per base per generation and a generation time (g) of 2.5 years were used for analysis.

### 2.9. Detection SNPs under Selective Sweep Analysis

To investigate the potential selective signals during *M. amblycephala* and *M. hoffmanni* dietary adaptation, genomic regions were scanned based on genome-wide calculations for selective sweeps by differentiation index (Fst), nucleotide polymorphism ratio (PiR), and extended haplotype homozygosity between populations (XP-EHH). vcftools software [27] was used to analyze the Fst and PiR values with a sliding window of 50 Kb and a step size of 10 Kb. For XP-EHH analysis, selscan v1.2.0 software [32] was applied and norm parameter used to normalize the original XP-EHH value, and the average value in the sliding window calculated according to the 50 Kb sliding window and 10 Kb step size after the standardized value. The common regions of Top 5% Fst, High/Low top 5% PiR, and High/Low top 5% XP-EHH were identified as the selected regions. For the *M. amblycephala* populations, 132 regions with a total length of 6.68 M contained 166 genes were identified from the comparison between *M. amblycephala* and *M. skolkovii*; 133 regions with a total length of 6.67 M containing 179 genes identified from the comparisons between *M. amblycephala* and *M. pellegrini*; 97 regions with a total length of 5.32 M containing 191 genes identified from the comparisons between *M. amblycephala* and *M. hoffmanni*. For the *M. hoffmanni* populations, 75 regions with a total length of 4.14 M containing 126 genes were identified from the comparisons between *M. amblycephala* and *M. hoffmanni*. Genes from these selective regions were subjected to a functional enrichment analysis of selected genes with Gene Ontology (GO) and Kyoto Encyclopedia of Genes and Genomes (KEGG) (DAVID, v6.8) [33]. Statistical significance was accessed by using a modified Fisher’s exact test and Benjamini correction for multiple testing. The calculated *p*-value was based on Bonferroni Correction and the functional terms or pathways with corrected *p*-value < 0.05 defined as significantly enriched functional terms.

### 2.10. RNA Isolation and Real-Time qPCR

In this study, the liver and spleen tissues from *M. amblycephala* in LZL and *M. hoffmanni* in QY populations were selected. The total RNA of these samples was extracted by Trizol reagent (Invitrogen, Carlsbad, CA, USA). The quality and quantity of RNA were detected by NanoDrop 2000 (Thermo Scientific, Waltham, MA, USA), agarose gel electrophoresis and a UV spectrophotometer (Thermo Fisher Scientific Inc., Waltham, MA, USA). Then 1 µg total RNA was reverse-transcribed into cDNA using cDNA Synthesis Kit (TaKaRa, Shiga, Japan). qPCR primers are listed in Table S2. We screened and compared three reference genes (*β-actin*, *Gapdh*, and *18S rRNA*), and found that *β-actin* was more stable than other reference genes (Figure S1 and Table S3). Thus, *β-actin* was used as an internal control. The expression pattern of genes was investigated by the real-time qPCR method with a quantitative thermal cycler (MyiQTM 2 Two Color Quantitative PCR Detection System, Bio-Rad, Hercules, CA, USA). Calculations for relative expression were conducted by the 2^−^^ΔΔCt^ method. Statistical analysis was performed with SPSS 16.0 software and results are reported as mean ± standard error (SE). The data were analyzed by independent sample *t*-test, and a *p*-value < 0.05 was considered to indicate a statistical significance.

## 3. Results

### 3.1. Genome Resequencing and Variation Calling

**Figure 1:**
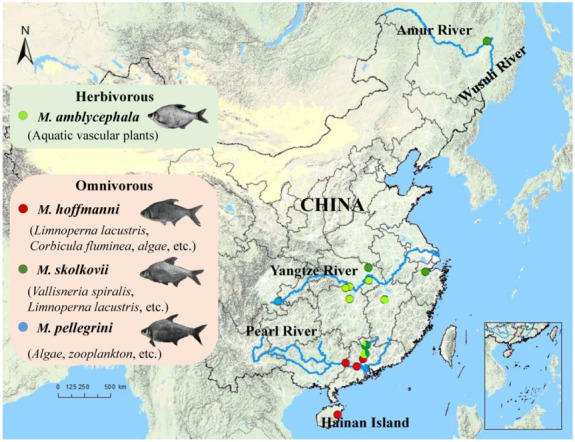
Geographic map indicating the distribution and feeding habits of the *Megalobrama* species in this study. Circles reflect the geographic regions where the samples were collected from. The blue lines represent the rivers.

A total of 180 *Megalobrama* fish representing different geographical populations in China were selected for genome resequencing (Figure 1, Table S4). To explore the phylogenetic relationships and evolutionary history of *Megalobrama*, all samples were sequenced to an average sequencing depth of ~17.43 × per individual yielding 94% sequencing coverage, an average Q20 of 97% and an average Q30 of 90% (Table S5). After filtering and quality control, the sequencing generated a total of 4.063 Tb raw databases and 3.483 Tb clean databases. We mapped all individuals to the *M. amblycephala* genome and detected 31,857,189 SNPs in the *Megalobrama* populations (Table S6).

### 3.2. Phylogeny and Population Structure Analysis

**Figure 2:**
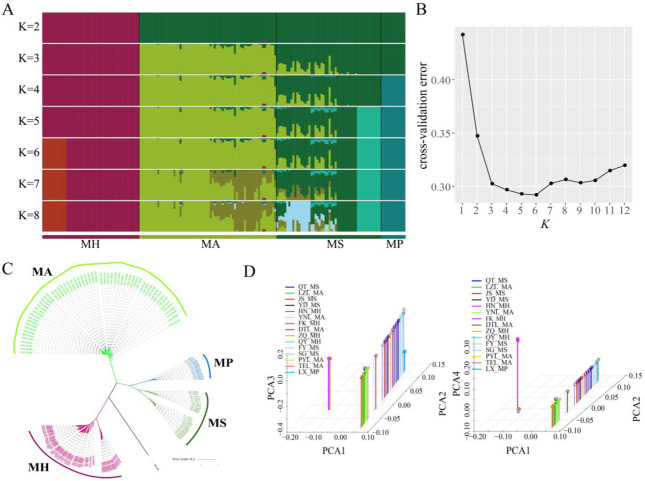
Phylogenetic analysis of different *Megalobrama* geographical populations. (**A**) Population genetic structure of 180 *Megalobrama* fish. The length of each colored segment represents the proportion of the individual genome inferred from ancestral populations (*K* = 2–8). The species name is at the bottom. (**B**) Cross-validation (CV) error for varying values of *K* in the admixture analysis. The minimum of estimated CV error on *K* = 6 suggests the most suitable number of ancestral populations. (**C**) Maximum-likelihood-based phylogenetic tree constructed using no admixed individuals. The scale bar represents pairwise distances between different individuals. Different colors represent different *Megalobrama* species. (**D**) Principal component analysis (PCA) of *Megalobrama* populations. Eigenvector 1, 2, 3, and 4 explained 45.06%, 18.03%, 4.00%, and 1.73% of the total variance, respectively. MH refers to *M. hoffmanni*, MA to *M. amblycephala*, MS to *M. skolkovii*, MP to *M. pellegrini*.

Admixture analysis was used to cluster individuals based on all high-confidence SNP sites. When ancestry components (*K*) = 6, six geographically distributed ancestral components (*K*) were labeled as: HN and Pearl River *M. hoffmanni* populations; *M. amblycephala* populations; FY and other *M. skolkovii* populations; and *M. pellegrini* populations (Figure 2A). This finding was supported by cross-validation (when *K* = 6, the cross-validation error was minimum) and was consistent with the phylogeny and biogeographic distribution (Figure 2B). Using the *Danio rerio* as an outgroup, a phylogenetic tree was constructed by the maximum likelihood method based on all non-admixed individuals. All of the samples were clustered into three branches. Initially, *M. amblycephala* and *M. hoffmanni* were clustered into a single branch and *M. skolkovii* and *M. pellegrini* clustered into one branch (Figure 2C). The findings revealed a close genetic affinity between *M. skolkovii* and *M. pellegrini* groups whereas the *M. hoffmanni* population showed genetic divergence from the other *Megalobrama* groups. Additionally, principal components analysis (PCA) provided supporting evidence for these groupings (Figure 2D). The first four principal components divided the *Megalobrama* populations into six subgroups. Taken together, the results provided compelling evidence that *Megalobrama* species from various geographical distributions can be divided into six different subgroups.

### 3.3. M. amblycephala Introgression into M. skolkovii

**Figure 3:**
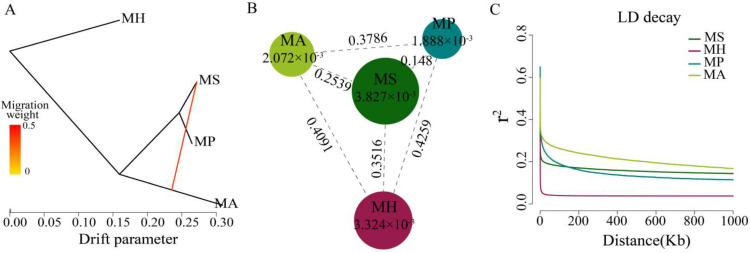
Population genetic analysis of *Megalobrama* species. (**A**) Gene flow analysis of *Megalobrama* species. Arrows indicate migration events that occur between populations. The heat map indicates migration weight. (**B**) Nucleotide polymorphism (π), and differentiation index (Fst) of the four *Megalobrama* species. The largest circle represents the large π value, and the longer line segment represents the large Fst value. MH refers to *M. hoffmanni*, MA to *M. amblycephala*, MS to *M. skolkovii*, MP to *M. pellegrini*. (**C**) Decay of linkage disequilibrium (LD) patterns for the four *Megalobrama* species inferred by the phylogenetic trees.

In this study, we used Treemix to confirm the gene flow from *M. amblycephala* to *M. skolkovii*. Using *M. hoffmanni* as the outgroup, among the four *Megalobrama* species, only one migration event was inferred from the *M. amblycephala* to the *M. skolkovii* with approximately 50.77% DNA gene flow from *M. amblycephala* to *M. skolkovii* (Figure 3A). Moreover, the migration patterns of these two species’ geographic populations demonstrated that LZL, DTL, TEL, YNL, and PYL *M. amblycephala* populations introgressed into the FY, SG, QT, and JS *M. skolkovii* populations. Notably, the migration weight of *M. amblycephala* introgressed into QT and JS populations was greater than that of the FY and SG populations (Figure S2).

### 3.4. Linkage Disequilibrium and Genetic Diversity

The genetic diversity (π) of *M. skolkovii* and *M. hoffmanni* were estimated to be 3.827 × 10^−3^ and 3.324 × 10^−3^, respectively, which was relatively high in comparison to *M. amblycephala* (2.072 × 10^−3^) and *M. pellegrini* (1.888 × 10^−3^). The low genetic differentiation (Fst) between *M. skolkovii* and *M. pellegrini* (0.148), and the high Fst between *M. hoffmanni* and *M. amblycephala* (0.4091), *M. skolkovii* (0.3516), and *M. pellegrini* (0.4259) (Figure 3B) are consistent with the phylogeny analysis. Between the four *Megalobrama* species, linkage disequilibrium (LD) and correlation coefficient (*r*^2^) values were calculated. The decay of LD reached half the maximum average *r*^2^ at a distance of 24 Kb, 4 Kb, 1.8 Kb, and 0.2 Kb for *M. amblycephala*, *M. pellegrini*, *M. skolkovii*, and *M. hoffmanni*, respectively (Figure 3C). Therefore, *M. skolkovii* and *M. hoffmanni* displayed a faster LD decay rate than *M. amblycephala* and *M. pellegrini*.

### 3.5. Demographic History of Megalobarma Species and Species Delimitation

**Figure 4:**
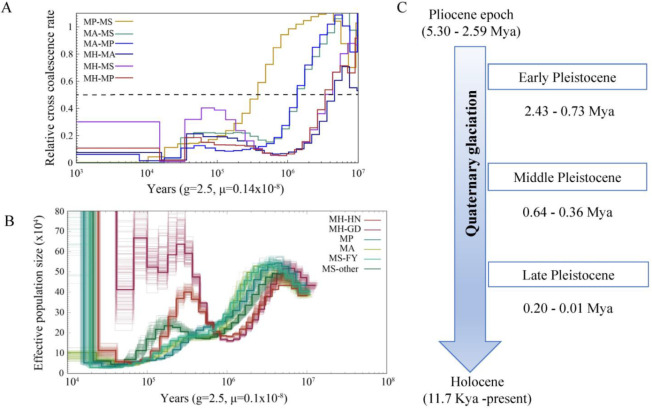
Demographic history of *Megalobrama* species. (**A**) Relative cross coalescence rates (CCR) between *Megalobrama* populations. When the two populations are completely mixed, the CCR is close to one. When they are completely split, the CCR is close to zero. The dotted line indicates that the CCR is 0.5. MP, MS, MA, and MH refer to *M. pellegrini*, *M. skolkovii*, *M. amblycephala*, and *M. hoffmanni*. g (generation time) = 2.5 years; μ (neutral mutation rate per generation) = 0.14 × 10^−8^. (**B**) PSMC model estimates changes in the effective population size over time, representing variation in inferred Ne dynamics. The undulating broken line in the figure is the estimated effective population size of each population in the evolutionary history. The time axis is not divided into deciles. μ = 0.1 × 10^−8^. MH-HN, MH-GD, MP, MA, MS-FY, and MS-other refer to the Hainan population of *M. hoffmanni*, Pearl River populations of *M. hoffmanni, M. pellegrini*, *M. amblycephala* population, Fuyuan population of *M. skolkovii*, and other *M. skolkovii* populations. (**C**) Timeline of Quaternary glaciation. Mya = million years ago.

The split times based on the relative cross-coalescent rates (RCCR) among the four *Megalobrama* species reached 0.5 suggesting a split between *M. hoffmanni* and other species 3–5 Mya. The cross-coalescence analysis suggested a decline to 0.5 between *M. amblycephala* and *M. skolkovii* or *M. pellegrini* at ~1.3 Mya, and a decline to 0.5 between *M. skolkovii* and *M. pellegrini* around 0.3–0.4 Mya (Figure 4A). The PSMC method used to reconstruct the demographic history of six *Megalobrama* subgroups indicated that the effective population size (*Ne*) peak of *M. amblycephala* and *M. pellegrini* was 2.5 Mya and 4 Mya, respectively, compared to 3–4 Mya of the *Ne* peak for the *M. skolkovii* subgroups. After that, the FY *M. skolkovii* subgroup continued to shrink, whilst the *Ne* curves of other *M. skolkovii* subgroup split at 0.2 Mya expanded (Figure 4B). Moreover, the two *Ne* peaks of *M. hoffmanni* occurred at 0.3 Mya and 5 Mya, respectively, during the middle pleistocene geological period (Figure 4C). Interestingly, the split of *Ne* curves of the two *M. hoffmanni* subgroups occurred about 0.4 Mya, indicating a population divergence at this time. The ancestral reconstruction analysis revealed two different biogeographic evolutionary processes to investigate the ancestral distribution of genus *Megalobrama*. According to the BBM analysis, the genus *Megalobrama* was originally distributed in the Pearl River and then spread to Hainan Island and Northern China. However, the analysis based on the S-DIVA and DEC models showed that the *Megalobrama* ancestors originally inhabited the Pearl River and Yangtze River, before spreading to the Amur and Wusuli Rivers (Figure S3).

### 3.6. Selective Sweeps for Dietary Adaptation of Megalobrama

**Figure 5:**
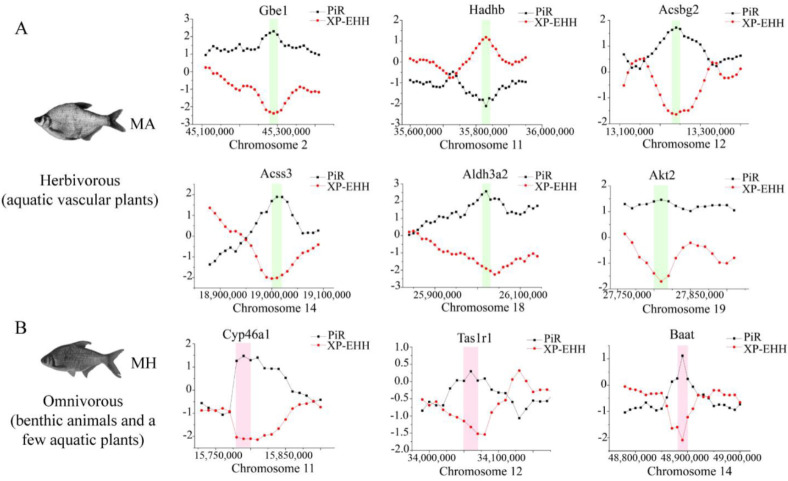
Genome-wide inference of selection sweeps on chromosomes during the diet adaptation of *M. amblycephala* (**A**) and *M. hoffmanni* (**B**). MA and MH refer to *M. amblycephala* and *M. hoffmanni*. *Gbe1*, *Akt2*, and *Aldh3a2* were identified from the comparison between *M. amblycephala* and *M. skolkovii*, *Acss3*, and *Acsbg2* from the comparison between *M. amblycephala* and *M. pellegrini, Hadhb* from the comparison between *M. amblycephala* and *M. hoffmanni,* and *Cyp46a1*, *Tas1r1*, and *Baat* from the comparison between *M. hoffmanni* and *M. amblycephala*. The black curve indicates the nucleotide polymorphism ratio (PiR) analysis, and red curve indicates extended haplotype homozygosity between populations (XP-EHH) analysis. The green and pink boxes represent the position of the selected genes on the chromosomes.

**Figure 6:**
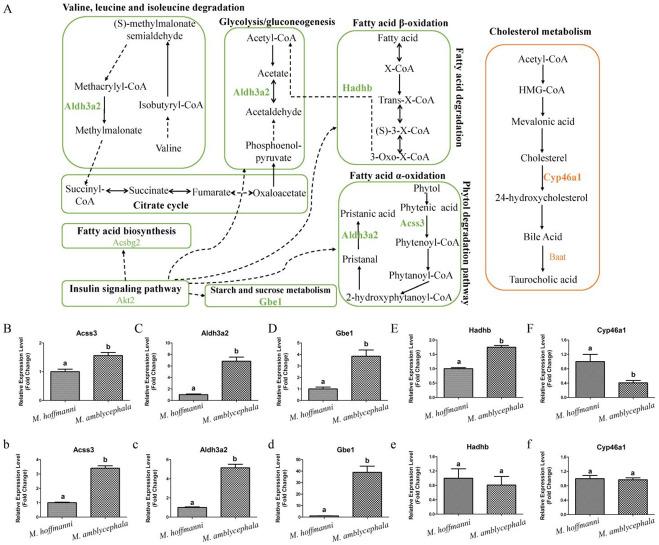
Metabolism pathways and expression pattern of candidate genes. (**A**) Candidate genes of *M. amblycephala* and *M. hoffmanni* enriched in the metabolism pathways. The lines and selected genes in *M. amblycephala* and *M. hoffmanni* are indicated in green and pink, respectively. The dashed lines used to connect KEGG pathways represent indirect relationships. (**B**–**F**) The expression pattern of candidate genes (*Acss3*, *Aldh3a2*, *Gbe1*, *Hadhb*, and *Cyp46a1*) in the liver tissue of *M. amblycephala* and *M. hoffmanni*. (**b**–**f**) The expression pattern of candidate genes (*Acss3*, *Aldh3a2*, *Gbe1*, *Hadhb*, and *Cyp46a1*) in the spleen tissue of *M. amblycephala* and *M. hoffmanni*. Different letters indicate a significant difference (*p* < 0.05).

To investigate the potential selective signals during *M. amblycephala* dietary adaptation, we scanned the genomic regions based on genome-wide calculations for selective sweeps by estimating Fst, PiR, and XP-EHH values. Candidate genes were discovered in the common region of top 5% Fst, High/Low top 5% PiR, and High/Low top 5% XP-EHH, inferred from the comparisons between *M. amblycephala* and the other three species (Figure 5A, Figures S4 and S5A). Fatty acid degradation, glycerolipid metabolism, beta-alanine metabolism, arginine and proline metabolism, histidine metabolism, insulin secretion, and lysine degradation, among other metabolic processes were associated with a significant portion of the candidate genes, according to GO and KEGG analyses (Figure S6). *M. amblycephala* mainly feeds on high-fiber and low-energy aquatic vascular plants. The candidate genes of *M. amblycephala* were enriched in fatty acid degradation and corresponding upstream and downstream pathways. For instance, *Aldh3a2* and *Acss3* genes in fatty acid α-oxidation, *Hadhb* gene in fatty acid β-oxidation, *Akt2* gene in insulin signaling pathway, *Aldh3a2* gene in glycolysis/gluconeogenesis, *Gbe1* gene in starch and sucrose metabolism, *Acsbg2* gene in fatty acid biosynthesis, and *Aldh3a2* gene in valine, leucine, and isoleucine degradation (Figure 6A and Table S7). Moreover, these genes exhibited a high expression pattern in the liver and/or spleen tissue of *M. amblycephala* compared with *M. hoffmanni* (Figure 6B–E,b–e).

Among the three omnivorous *Megalobrama* species, the food composition of *M. hoffmanni* is rich in zoobenthos. The common regions of top 5% Fst, High top 5% PiR, and Low top 5% XP-EHH were the selected regions inferred from a comparison of *M. hoffmanni* and *M. amblycephala*, which covered a total of 75 selected regions with a length of 4.14 Mb and including 126 genes in *M. hoffmanni*. These genes were found to be abundant in cellular and metabolic processes including taste transduction, fatty acid elongation, biosynthesis of unsaturated fatty acids, and biosynthesis of amino acids according to GO and KEGG enrichment analysis (Figure S7). Interestingly, we found some candidate genes (*Cyp46a1* and *Baat*) involved in cholesterol metabolism (Figure 5B, Figure 6A, Figure S5B and Table S7). The results indicated that compared with *M. amblycephala*, the *Cyp46a1* gene was highly expressed in the liver of *M. hoffmanni*, but not in the spleen (Figure 6F,f). Furthermore, functional annotation indicated that the umami taste receptor gene *Tas1r1* was abundant in the *M. hoffmanni* sensory system.

## 4. Discussion

Until now, the main resources available to investigate the evolution of *Megalobrama* species were morphological and mt DNA analyses. There have been some controversies over the *Megalobrama* species in terms of phylogenetic relationship and species boundaries [4,7,8]. Our genomic population datasets revealed that all *Megalobrama* populations could be separated into six subgroups based on the geographical isolation, with each of *M. hoffmanni* and *M. skolkovii* divided into two subgroups. *M. skolkovii* was more closely related to *M. pellegrini* than *M. amblycephala* and *M. hoffmanni*, and *M. hoffmanni* clustered apart from other *Megalobrama* species, showing a high nucleotide diversity and fast LD decay rate in the geographic groups. This finding contributes to resolving the fundamental issue of the phylogenetic relationship of the four *Megalobrama* species. Recently, artificial introductions have hampered the protection of the *Megalobrama* genus germplasm resources, particularly *M. amblycephala* and *M. skolkovii*. Treemix validated gene flow from *M. amblycephala* to *M. skolkovii*. We speculate that, as a result of the introduction of *M. amblycephala*, introgression increased the nucleotide polymorphism of *M. skolkovii*. It implies that introgression may provide an important source of genetic variation in *M. skolkovii* populations.

The glacial epoch saw a dip in sea-level of 80–130 m compared to the present, wide stretches of the continental shelf emerged and a land bridge formed across the South China Sea [34]. With this connection, the Pearl River *M. hoffmanni* population likely expanded its distribution range, resulting in the Hainan population. After the glaciers subsided, sea-level rise triggered a phylogeographic break which differentiated the freshwater fish populations living on Hainan Island from those on mainland [35]. It has been reported that the South China Sea transgression during the late Pleistocene interglacial or post-glacial period may have reduced the habitat territory for freshwater fish, resulting in population contraction [36]. Consequently, the effective population size of *M. hoffmanni* in the Pearl River declined throughout this time period. The two critical features of the Quaternary Ice Age are the change of sea level and the existence of high mountains, especially the uplift of the Qinghai-Tibet Plateau, the Yunnan-Guizhou Plateau and the western high mountains, and the large-scale crustal subsidence in the east that has caused gradual disintegration of most water systems [37]. The extant distribution of the freshwater fish fauna in China developed during the elevation of the Qinghai-Tibet Plateau from the late Tertiary to the early Quaternary period [38]. With the uplift of the Qinghai-Tibet Plateau, the original lake basins in the middle and lower reaches of the Yangtze River progressively disintegrated, and the closed lake basins merged, establishing the initial embryonic shape of the Yangtze River. *M. pellegrini*, *M. amblycephala*, and *M. skolkovii* populations in the Yangtze River experienced population contraction due to changes in terrain. However, the increase in the *M. skolkovii* population in the Qiantang River, adjacent to the East China Sea, may have been caused by a sea-level reduction during the glacial period.

To adapt to their habitat environment, *Megalobrama* species have evolved and adapted to different food compositions. *M. amblycephala*, for example, feeds mainly on high-fiber, low-energy aquatic plants. The candidate genes in selective regions of *M. amblycephala* were associated with lipid metabolism and also the corresponding upstream and downstream pathways. For instance, the *Hadhb* gene encodes enzymes involved in the final reactions that catalyze the β-oxidation of fatty acids [39]. Aldh3a2 is a typical fatty aldehyde dehydrogenase that catalyzes the oxidation of long-chain aldehydes produced by lipid metabolism [40]. It has been shown that adaptive alterations in fatty acids β-oxidation and lipid metabolism can increase the amount of ATP required by the foregut fermenters [41]. Additionally, plant cell walls (such as cellulose and hemicellulose) can be degraded to produce short-chain fatty acids, which are high in energy and serve as the primary source of energy for foregut fermenters [42]. Moreover, *Aldh3a2* is implicated in the phytol degradation pathway and plays a dominant role in the oxidation of pristine to pristanic acid [43]. Phytol is a side chain of the plant pigment chlorophyll that following consumption by mammals, can be metabolized in the body to form phytanic acid and norphytanoic acid by α-oxidation [44]. Phytol and its metabolites are major sources of energy in fish oxidative metabolism. As a result, *M. amblycephala* may have developed a complete fatty acid degradation pathway associated with α-oxidation and β-oxidation of fatty acids to enhance energy metabolism and effectively absorb and utilize scarce nutrients.

The diet of *M. hoffmanni* consists primarily of benthic animals and little aquatic vegetation. Interestingly, we discovered that the candidate genes in the *M. hoffmanni* selective regions were associated with cholesterol metabolism. Cholesterol is a precursor of bile acids, which are required for the absorption of cholesterol, fats, and lipophilic vitamins in the intestine [45]. Bile acids have been shown to aid in the digestion and absorption of dietary lipid [46]. Cyp46a1 binds to cholesterol and catalyzes the synthesis of the oxysterol 24 (S)-hydroxycholesterol [47]. Furthermore, Baat is the terminal enzyme for cholesterol synthesis of bile salt, which catalyzes the coupling of taurine or glycine with bile acid coenzyme, a thioester to form bile acid N-acetamidomide [48]. A previous study found that serum cholesterol levels in mammals on a high-fat diet are high, while levels in those on a high-protein diet are low [49]. Cholesterol is the most common steroid component found in animal food. It is commonly found in animal cells [50]. We found that the *Cyp46a1* gene was highly expressed in *M. hoffmanni* compared with *M. amblycephala*. Besides, the sensory system gene *Tas1r1* was inferred to be positively selected in the *M. hoffmanni* populations. Tas1r1, a member of the Tas1r family can bind to Tas1r3 to form the umami taste receptor. Previous studies have indicated that the umami taste receptor gene *T1R1* is missing in the genome of giant pandas that feed on bamboo, but multiple copies of the gene *TIRI* detected in the genomes of omnivorous (*Oryzias latipes* and *Gasterosteus aculeatus*), and carnivorous (*Gadus morhua* and *Cynoglossus semilaevis*) fish [11,51]. Taken together, we speculate that during its adaption to the habitat in the Pearl River, *M. hoffmanni* may have developed unique metabolic pathways for animal food.

## 5. Conclusions

Population genomic research provided new insights into phylogenetics, population structure, demographic history reconstruction, and feeding habits of the genus *Megalobrama*. The screening of selective genomic regions related to the *Megalobrama* diet elucidated a significant role in the environmental adaptation mechanisms of the four *Megalobrama* species. Importantly, this study has provided a valuable genomic resource for future genetic studies that can contribute to improvement of the *Megalobrama* species using genome-assisted breeding.

## Data Availability

The sequence data of *Megalobrama* genome resequencing involved in this study have been deposited in NCBI with the BioProject accession number PRJNA756243.

## Supplementary Materials

The following supporting information can be downloaded at: https://www.mdpi.com/article/10.3390/biology11020186/s1, Figure S1: Detection of stability of reference genes (*β-actin*, *Gapdh*, and *18S rRNA*). Figure S2: Gene flow analysis among different geographical *Megalobrama* populations. Figure S3: Ancestral area reconstruction based on BBM model (A), DEC model (B), and S-DIVA model (C) with RASP. Figure S4: Genome-wide detection of selection sweeps on chromosomes during the diet adaptation of *M. amblycephala*. Figure S5: Genome-wide screening for *M. amblycephala* (A) and *M. hoffmanni* (B) diet-associated selective sweeps estimating differentiation index (Fst). Figure S6: Gene Ontology (GO) analysis of candidate genes of *M. amblycephala* in MA-MH (A), MA-MS (B) and MA-MP (C) groups, and Kyoto Encyclopedia of Genes and Genomes (KEGG) analysis of these genes screened from MA-MH (a), MA-MS (b) and MA-MP (c) groups. Figure S7: Gene Ontology (GO) (A) and Kyoto Encyclopedia of Genes and Genomes (KEGG) (B and C) analyses of candidate genes in *M. hoffmanni* screened by selective sweeps inferring from the comparison between *M. hoffmanni* and *M. amblycephala*. Table S1: Mapping statistics of samples in whole genome resequencing. Table S2: Primers of the described sequences. Table S3: Comparison of stability of reference genes. Table S4: The distribution and number of samples in this study. Table S5: Data information of genome resequencing. Table S6: Variation information of samples in this study. Table S7: Candidate genes and enriched pathways in selective sweep analysis.
