# Supplementary material for: Population Genomics of Megalobrama Provides Insights into Evolutionary History and Dietary Adaptation
Source: Biology (Basel). 2022 Jan 25;11(2):186. doi: 10.3390/biology11020186 (PMC8869164; doi:10.3390/biology11020186)
Supplement: Supplementary file 1 [file biology-11-00186-s001.zip › biology-1429028-supplementary.pdf]

Supplementary materials

Article

Population Genomics of *Megalobrama* Provides Insights into Evolutionary History and Dietary Adaptation

Jing Chen [1,2], Han Liu [1,2,*], Ravi Gooneratne [3], Yao Wang [1,2], and Weimin Wang [1,2,*]

[1] Key Lab of Agricultural Animal Genetics, Breeding and Reproduction of Ministry of Education, College of Fisheries, Huazhong Agricultural University, Wuhan 430070, China; cjing24511@163.com (J.C.); othnielartorious@gmail.com (Y.W.)
[2] Key Lab of Freshwater Animal Breeding, Ministry of Agriculture and Rural Affairs, College of Fisheries, Huazhong Agricultural University, Wuhan 430070, China;
[3] Faculty of Agriculture and Life Sciences, Lincoln University, Lincoln 7647, New Zealand; ravi.gooneratne@lincoln.ac.nz
* Correspondence: liuhan@mail.hzau.edu.cn (H.L.); wangwm@mail.hzau.edu.cn (W.W.)

Supplementary figures

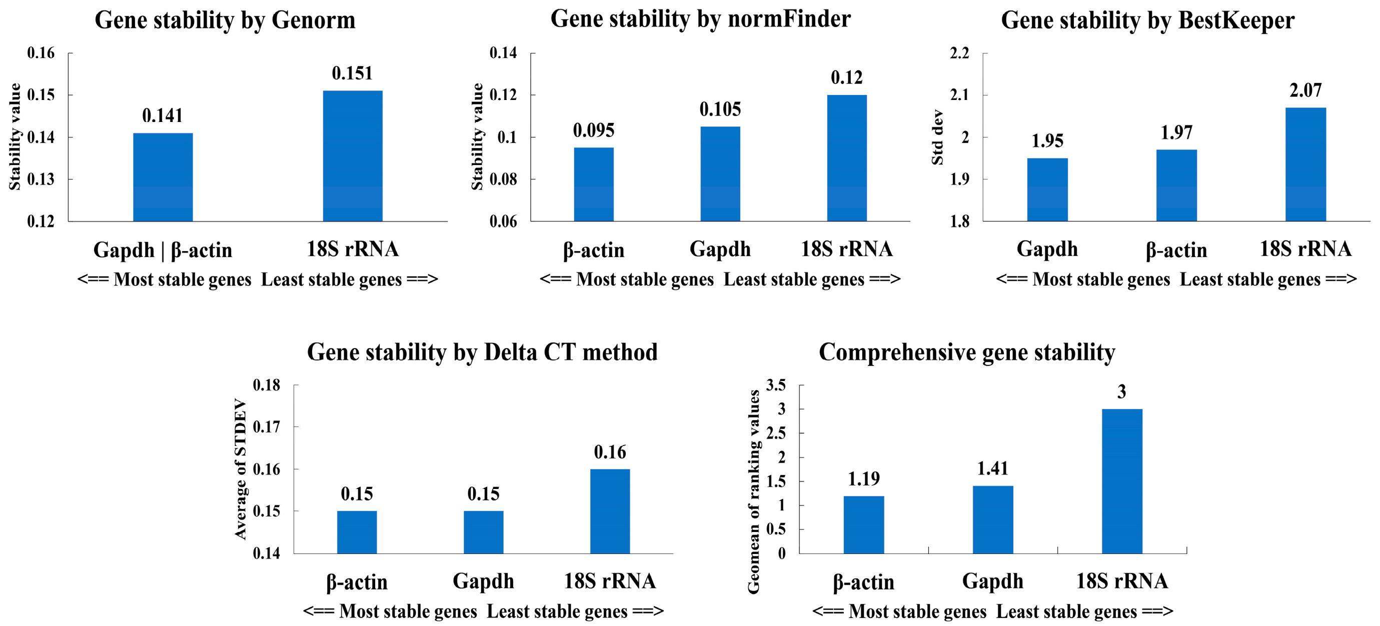

Figure S1. Detection of stability of reference genes (*β-actin*, *Gapdh*, and *18S rRNA*).

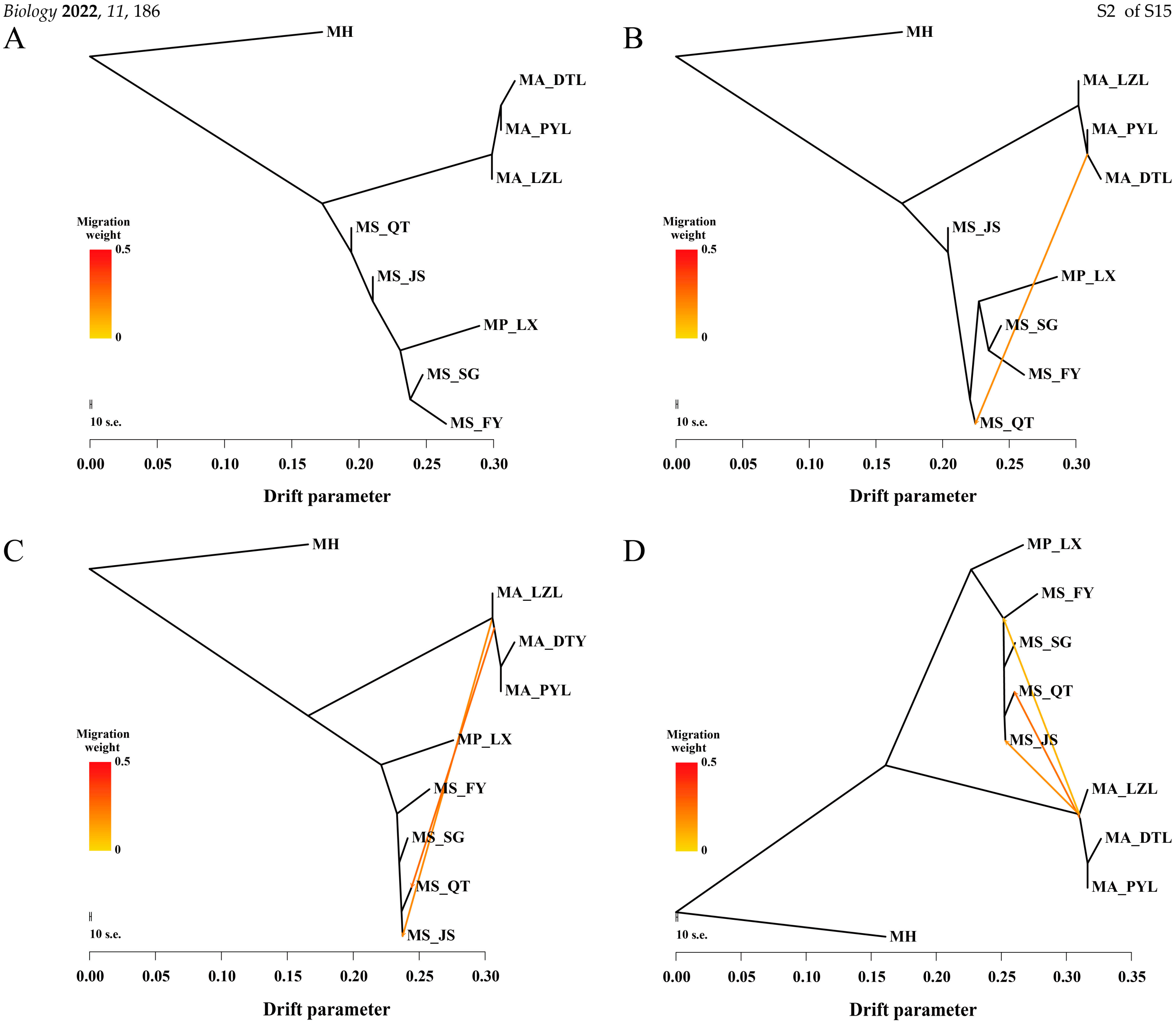

Figure S2. Gene flow analysis among different geographical *Megalobrama* populations. A-D represents that there are 0,1, 2, and 3 migration events between the population. Arrows indicate gene infiltration or migration events that occur between populations. The heat map indicates the weight of gene infiltration, the redr color indicates the greater the weight, each branch represents a population, and the length of the horizontal branch refers to each branch. The percentage of genetic drift that has occurred and the bottom axis represents drift parameters. DTY represents DTL, TEL, and YNL populations. MP, MS, MA, and MH refer to *M. pellegrini*, *M. skolkovii*, *M. amblycephala* and *M. hoffmanni*, respectively.

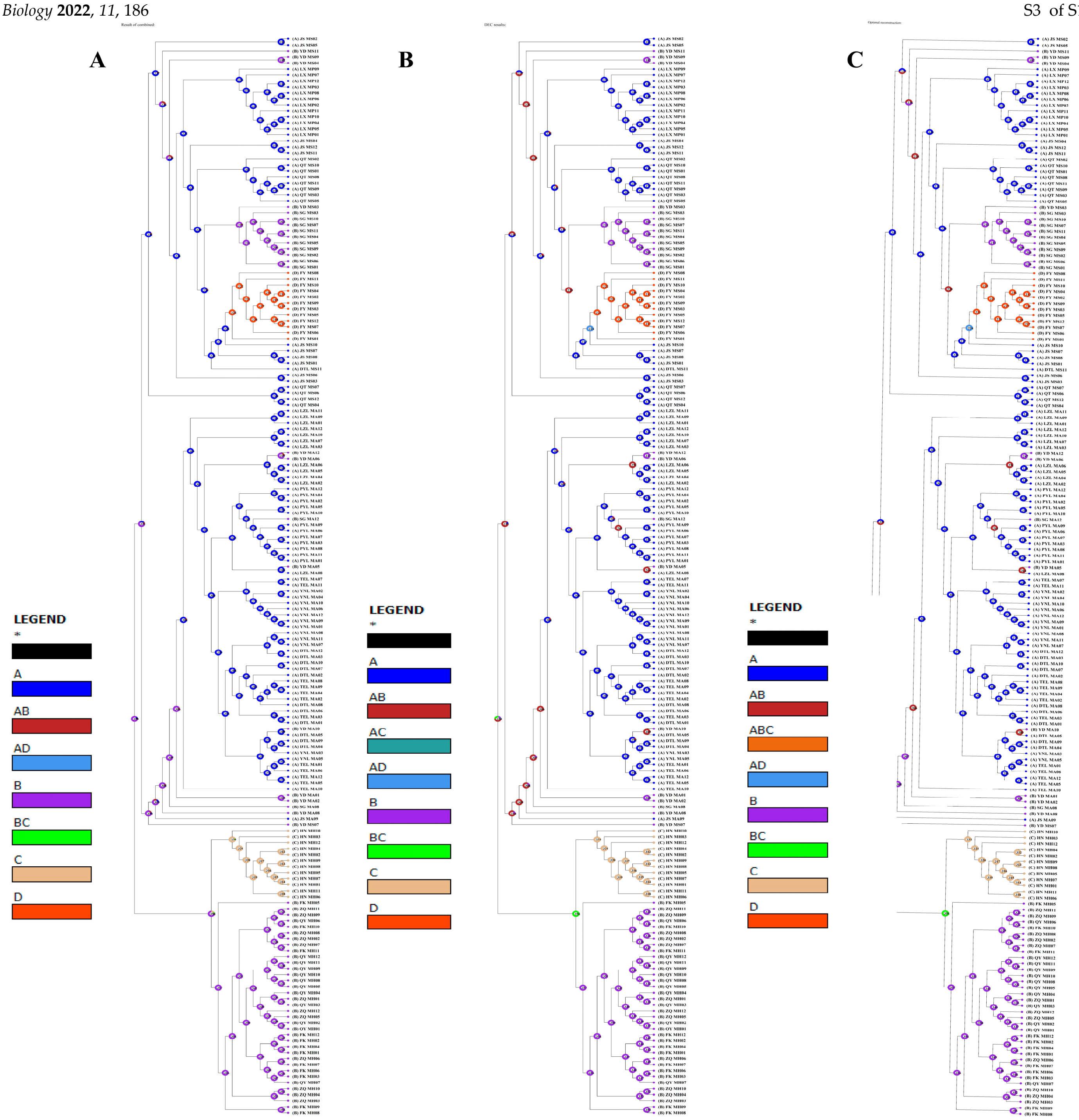

Figure S3. Ancestral area reconstruction based on BBM model (A), DEC model (B), and S-DIVA model (C) with RASP. The letter A represents the Yangtze River, the letter B represents the Pearl River, the letter C represents the Hainan Island, and the letter D represents the Heilongjiang and Wusuli River. MP, MS, MA, and MH refer to *M. pellegrini*, *M. skolkovii*, *M. amblycephala* and *M. hoffmanni*, respectively.

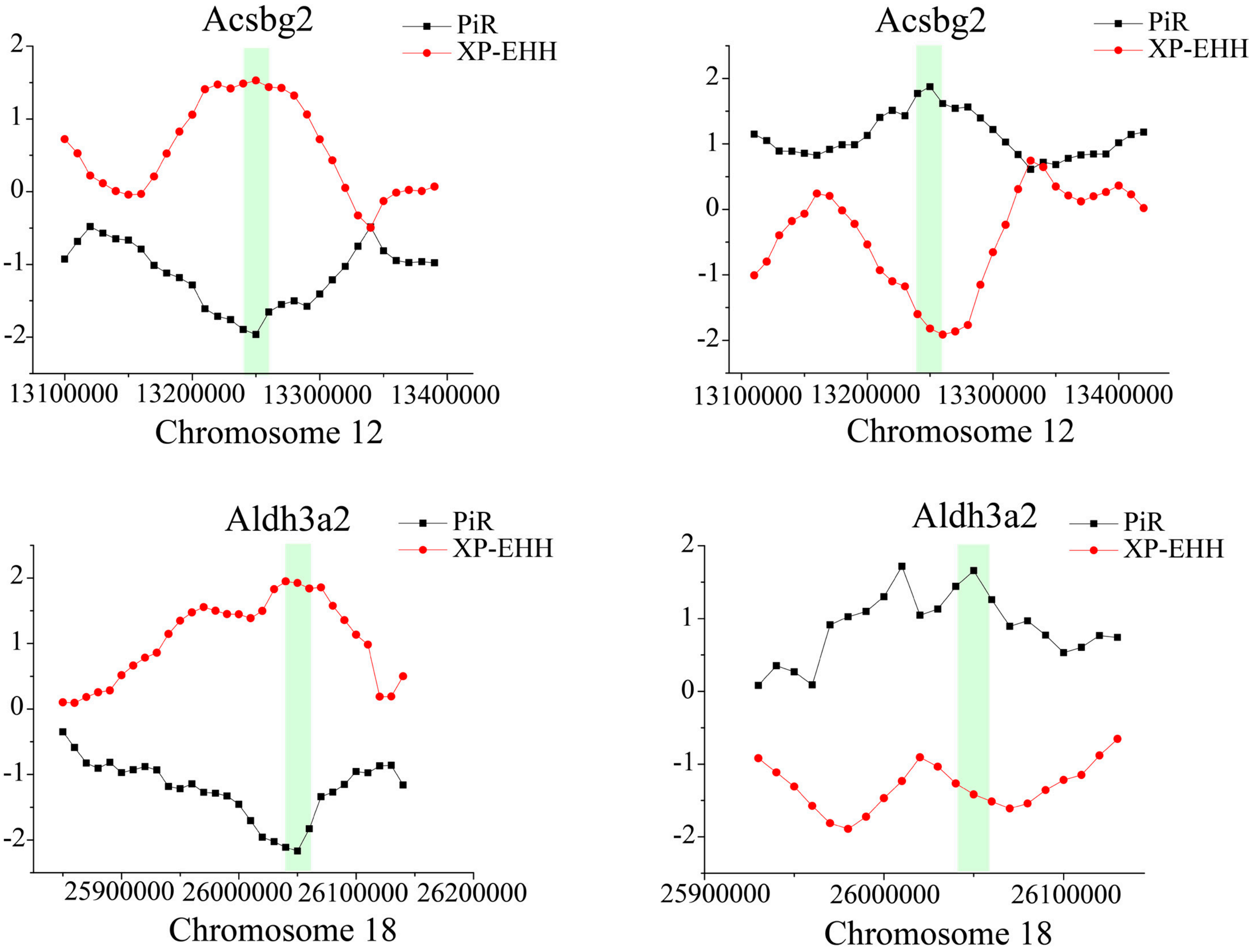

Figure S4. Genome-wide detection of selection sweeps on chromosomes during the diet adaptation of *M. amblycephala*. *Acsbg2* gene was identified from the comparisons between *M. amblycephala* and *M. hoffmanni* (or *M. skolkovii*), *Aldh3a2* gene was identified from the comparisons between *M. amblycephala* and *M. hoffmanni* (or *M. pellegrini*). The black curve indicates the nucleotide polymorphism ratio (PiR) analysis, and red curve indicates extended haplotype homozygosity between populations (XP-EHH) analysis.

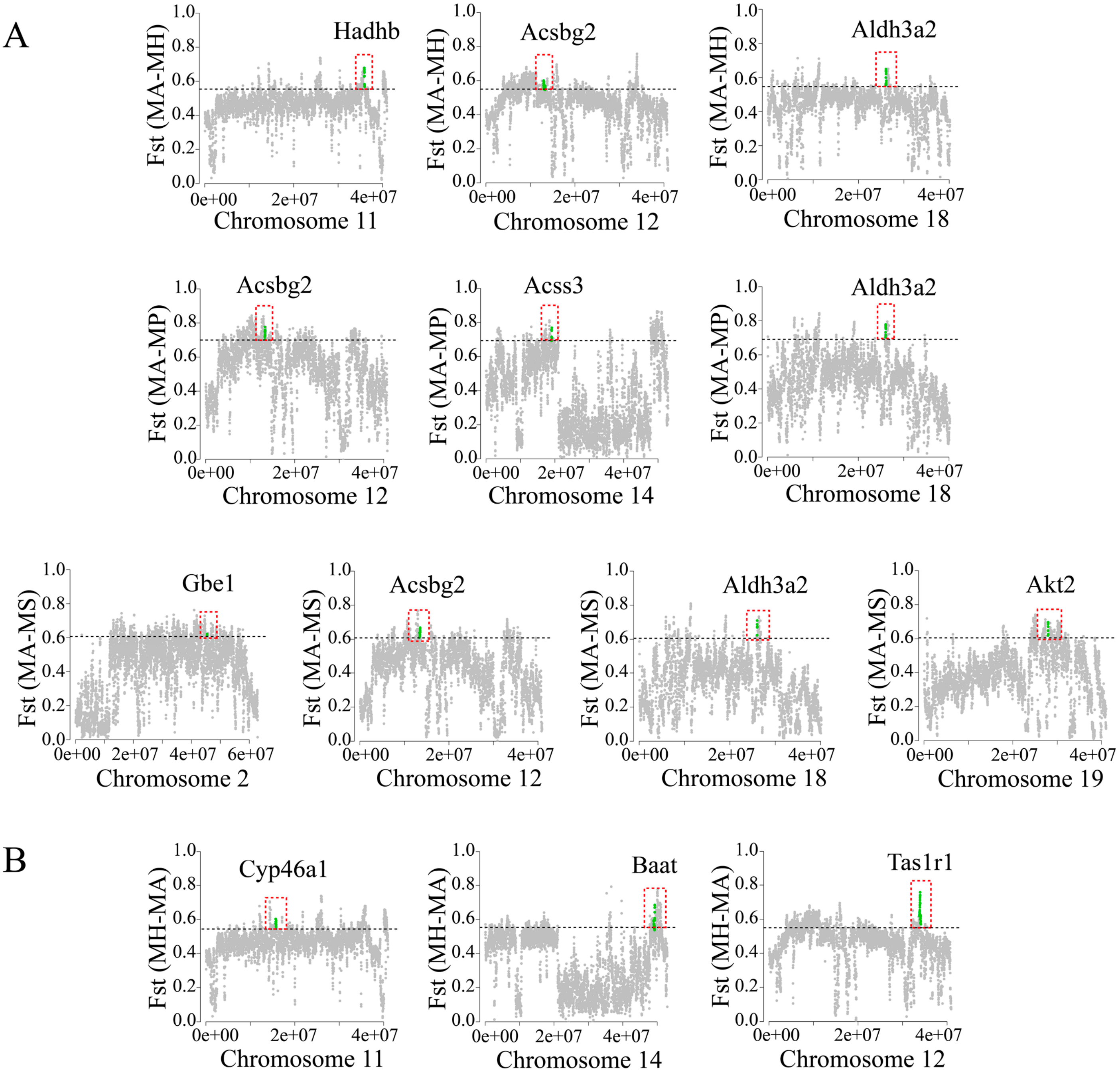

Figure S5. Genome-wide screening for *M. amblycephala* (A) and *M. hoffmanni* (B) diet-associated selective sweeps estimating differentiation index (Fst). The green dots represent the position of representative candidate genes on chromosomes. The black horizontal dashed line in Manhattan plots showed the significance threshold of the top 5% Fst. MP, MS, MA, and MH refer to *M. pellegrini*, *M. skolkovii*, *M. amblycephala* and *M. hoffmanni*, respectively.

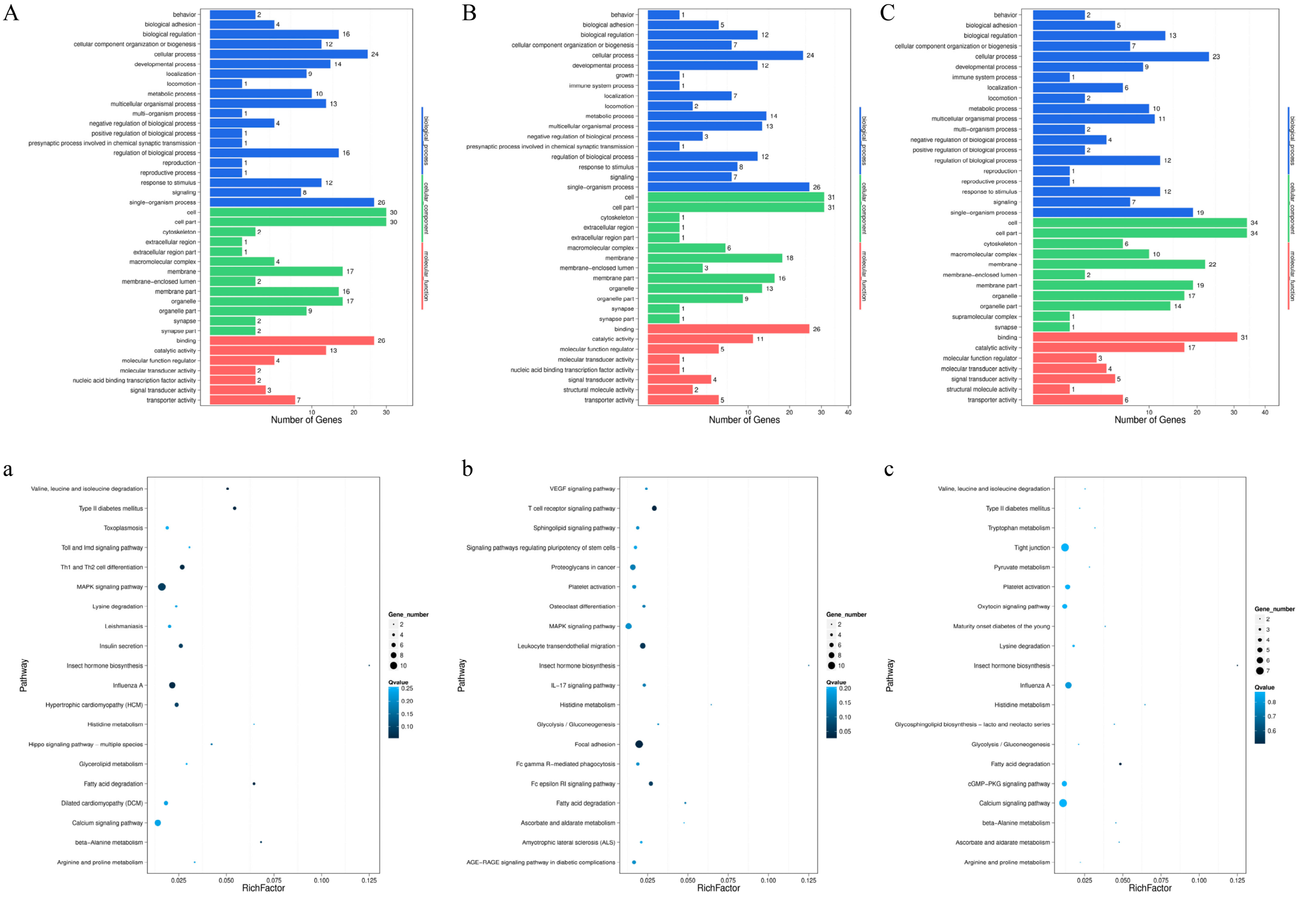

Figure S6. Gene Ontology (GO) analysis of candidate genes of *M. amblycephala* in MA-MH (A), MA-MS (B) and MA-MP (C) groups, and Kyoto Encyclopedia of Genes and Genomes (KEGG) analysis of these genes screened from MA-MH (a), MA-MS (b) and MA-MP (c) groups. MP, MS, MA, and MH refer to *M. pellegrini*, *M. skolkovii*, *M. amblycephala* and *M. hoffmanni*, respectively.

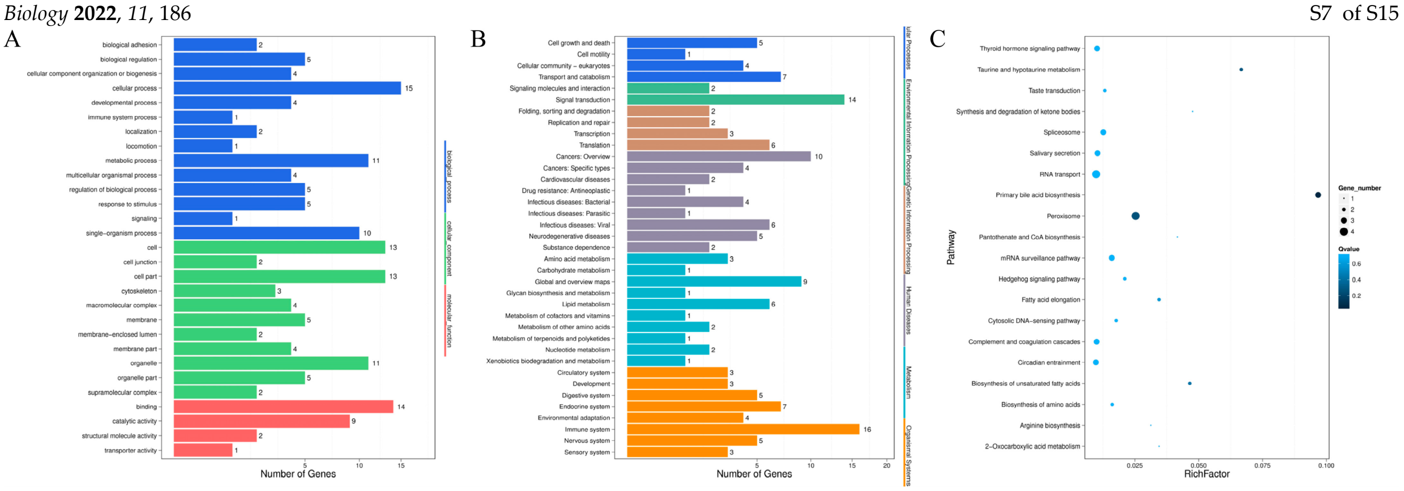

Figure S7. **Gene Ontology (GO) (A) and Kyoto Encyclopedia of Genes and Genomes (KEGG) (B and C) analyses of candidate genes in *M. hoffmanni* screened by selective sweeps inferring from the comparison between *M. hoffmanni* and *M. amblycephala*.**

Supplementary tables

Table S1. Mapping statistics of samples in whole genome resequencing.

Sample ID	Total reads(M)	Mapped reads(M)	Total bases(G)	Mapped bases(G)	Mapping rate (%)	Pair mapping rate (%)	Coverage rate at-least 1X (%)	Coverage rate at-least 5X (%)	Coverage rate at-least 9X (%)	Effective depth(X)
DTL_MA01	117.29	117.06	17.59	17.39	99.80	96.35	96.22	92.19	84.42	15.70
DTL_MA02	131.79	131.50	19.77	19.52	99.78	96.26	96.24	92.82	87.33	17.62
DTL_MA03	153.85	153.52	23.08	22.81	99.79	96.61	96.38	93.59	89.86	20.59
DTL_MA04	133.50	133.20	20.03	19.79	99.77	96.58	95.94	92.57	87.13	17.86
DTL_MA05	151.56	151.26	22.73	22.48	99.80	96.49	96.37	93.68	89.73	20.29
DTL_MA06	148.26	147.98	22.24	22.00	99.81	96.73	96.30	93.56	89.48	19.85
DTL_MA07	136.62	136.37	20.49	20.28	99.81	96.51	96.20	93.11	87.95	18.30
DTL_MA08	145.28	144.98	21.79	21.54	99.79	96.24	96.19	93.16	88.82	19.44
DTL_MA09	119.37	119.15	17.91	17.71	99.82	96.41	96.01	92.28	84.79	15.99
DTL_MA10	160.30	159.96	24.05	23.76	99.79	96.47	96.46	93.83	90.40	21.45
DTL_MS11	141.29	140.81	21.19	20.61	99.66	91.50	93.78	88.12	82.45	18.60
DTL_MA12	138.74	138.41	20.81	20.57	99.76	96.72	96.28	93.16	88.30	18.56
FK_MH01	124.20	123.56	18.63	17.79	99.49	87.90	91.05	81.82	74.15	16.06
FK_MH02	127.40	126.85	19.11	18.25	99.57	87.77	90.10	81.69	74.09	16.48
FK_MH03	106.38	105.85	15.96	15.23	99.51	86.88	89.83	80.25	69.95	13.75
FK_MH04	132.96	132.33	19.94	19.04	99.52	87.99	90.44	82.38	75.58	17.18
FK_MH05	123.63	122.88	18.54	17.68	99.40	87.51	90.17	81.60	73.90	15.96
FK_MH06	101.12	100.62	15.17	14.49	99.51	86.86	89.61	79.38	67.98	13.07
FK_MH07	129.66	126.01	19.45	17.92	97.19	85.31	90.28	81.75	74.29	16.17
FK_MH08	124.20	123.58	18.63	17.79	99.50	86.87	90.37	81.81	74.27	16.06
FK_MH09	127.73	127.09	19.16	18.30	99.49	86.81	90.48	82.06	74.80	16.51
FK_MH10	146.55	145.82	21.98	20.98	99.50	87.49	90.76	83.15	77.11	18.94
FK_MH11	111.77	111.11	16.77	16.00	99.41	86.55	90.16	80.92	71.68	14.44
FK_MH12	157.31	156.59	23.60	22.54	99.54	87.72	90.91	83.51	78.14	20.34
FY_MS01	116.84	116.42	17.53	17.06	99.64	92.32	92.86	86.36	78.10	15.40
FY_MS02	121.22	120.37	18.18	17.62	99.30	91.31	93.04	86.37	78.66	15.90
FY_MS03	132.46	131.82	19.87	19.28	99.52	91.96	93.34	87.39	80.93	17.40
FY_MS04	125.72	124.33	18.86	18.15	98.90	91.10	93.67	87.03	79.73	16.39
FY_MS05	138.54	137.16	20.78	20.08	99.00	91.13	93.09	87.49	81.75	18.12
FY_MS06	148.13	147.43	22.22	21.57	99.53	91.85	93.22	87.96	82.84	19.47
FY_MS07	140.74	139.97	21.11	20.49	99.46	91.63	93.47	87.76	82.14	18.49
FY_MS08	154.72	153.84	23.21	22.54	99.43	91.11	93.48	88.55	83.92	20.34
FY_MS09	152.13	151.25	22.82	22.13	99.42	91.52	93.54	88.35	83.54	19.98
FY_MS10	154.49	153.97	23.17	22.56	99.66	91.51	93.26	88.26	83.68	20.36
FY_MS11	147.19	146.59	22.08	21.48	99.59	91.41	93.35	88.12	83.02	19.39
FY_MS12	117.97	117.10	17.70	17.14	99.26	90.81	93.11	86.71	78.78	15.47
HN_MH01	145.67	144.98	21.85	20.90	99.52	87.37	89.77	82.31	76.66	18.86
HN_MH02	132.16	131.38	19.82	18.91	99.41	86.87	89.78	81.72	75.12	17.07
HN_MH03	119.43	118.85	17.91	17.12	99.52	86.95	89.54	80.79	73.12	15.45
HN_MH04	123.67	123.08	18.55	17.72	99.52	87.29	89.51	81.01	73.82	16.00
HN_MH05	128.50	127.91	19.27	18.42	99.54	87.23	89.68	81.36	74.62	16.63
HN_MH06	116.65	116.07	17.50	16.72	99.50	86.98	89.36	80.49	72.50	15.09
HN_MH07	134.12	133.44	20.12	19.22	99.49	87.14	89.72	81.59	75.28	17.35
HN_MH08	124.22	123.42	18.63	17.77	99.36	86.92	89.48	80.99	73.75	16.04
HN_MH09	132.18	131.53	19.83	18.95	99.51	87.05	89.78	81.52	74.78	17.10
HN_MH10	126.82	126.08	19.02	18.17	99.41	86.99	89.54	81.06	73.92	16.40
HN_MH11	108.91	108.01	16.34	15.52	99.17	85.82	89.28	79.91	70.80	14.01
HN_MH12	142.85	141.70	21.43	20.40	99.20	87.28	89.85	82.03	76.19	18.41
JS_MS01	110.89	110.50	16.63	16.17	99.64	92.14	93.12	85.90	75.92	14.59
JS_MS02	136.55	136.13	20.48	20.05	99.69	94.07	96.48	92.56	85.77	18.10
JS_MS03	136.93	136.51	20.54	20.06	99.69	93.31	95.23	90.51	84.08	18.10

JS_MS04	136.64	136.21	20.50	20.00	99.68	93.10	94.86	89.92	83.43	18.05
JS_MS05	124.26	123.91	18.64	18.25	99.72	94.01	96.16	91.60	83.21	16.47
JS_MS06	140.55	140.11	21.08	20.62	99.69	93.78	96.21	92.24	85.99	18.61
JS_MS07	125.31	124.89	18.80	18.27	99.66	91.82	93.28	86.93	79.73	16.49
JS_MS08	139.23	138.71	20.88	20.28	99.62	92.05	93.46	87.75	81.90	18.30
JS_MA09	164.82	164.38	24.72	24.29	99.74	95.31	96.70	93.76	89.59	21.92
JS_MS10	111.11	110.71	16.67	16.22	99.65	92.17	94.19	87.24	76.81	14.64
JS_MS11	119.27	118.75	17.89	17.37	99.56	91.59	93.34	86.75	78.72	15.68
JS_MS12	140.01	139.58	21.00	20.50	99.70	93.68	95.34	90.67	84.23	18.50
LX_MP01	126.88	126.37	19.03	18.48	99.60	90.02	92.59	86.26	79.50	16.68
LX_MP02	115.69	115.28	17.35	16.84	99.65	91.85	92.37	85.28	76.72	15.20
LX_MP03	108.35	107.91	16.25	15.77	99.60	91.03	92.10	84.65	75.01	14.23
LX_MP04	105.19	104.70	15.78	15.30	99.53	90.73	92.17	84.42	74.01	13.81
LX_MP05	106.09	105.66	15.91	15.43	99.60	90.69	92.16	84.48	74.33	13.92
LX_MP06	104.89	104.48	15.73	15.26	99.61	90.82	92.02	84.44	73.93	13.78
LX_MP07	103.56	103.17	15.53	15.07	99.63	91.06	91.98	84.12	73.38	13.61
LX_MP08	115.69	115.26	17.35	16.85	99.63	91.13	92.42	85.32	76.80	15.20
LX_MP09	113.62	113.18	17.04	16.53	99.62	91.06	92.37	85.26	76.61	14.92
LX_MP10	117.12	116.68	17.57	17.05	99.62	90.91	92.24	85.33	77.19	15.38
LX_MP11	122.85	122.31	18.43	17.87	99.56	91.49	93.23	85.90	78.58	16.13
LX_MP12	141.55	141.00	21.23	20.60	99.61	91.23	93.53	86.98	81.37	18.60
LZL_MA01	138.43	138.10	20.76	20.51	99.76	96.21	96.61	93.28	87.99	18.51
LZL_MA02	130.11	129.85	19.52	19.31	99.80	96.45	96.24	92.72	86.82	17.43
LZL_MA03	153.63	153.32	23.05	22.76	99.80	96.05	96.49	93.43	89.17	20.54
LZL_MA04	146.92	146.62	22.04	21.80	99.79	96.29	96.24	93.19	88.92	19.67
LZL_MA05	134.75	134.49	20.21	19.99	99.80	96.32	96.39	93.13	87.65	18.04
LZL_MA06	160.02	159.72	24.00	23.74	99.81	96.39	96.64	93.84	90.20	21.42
LZL_MA07	117.47	117.22	17.62	17.40	99.78	95.81	96.54	92.29	83.76	15.71
LZL_MA08	157.34	156.92	23.60	23.31	99.73	96.43	96.83	93.96	90.19	21.04
LZL_MA09	128.48	128.22	19.27	19.04	99.79	95.98	96.13	92.41	86.21	17.19
LZL_MA10	105.81	105.55	15.87	15.68	99.75	96.18	96.39	91.30	80.16	14.15
LZL_MA11	131.56	131.27	19.73	19.50	99.78	96.27	96.33	92.77	86.90	17.60
LZL_MA12	103.87	103.65	15.58	15.40	99.79	96.35	95.95	90.76	79.19	13.90
PYL_MA01	105.74	105.52	15.86	15.68	99.79	96.68	95.56	90.81	80.39	14.15
PYL_MA02	102.85	102.60	15.43	15.23	99.76	96.34	95.97	90.69	78.79	13.74
PYL_MA03	100.39	100.14	15.06	14.85	99.75	96.24	95.90	90.27	77.35	13.41
PYL_MA04	104.79	104.56	15.72	15.53	99.78	96.48	95.98	91.12	79.99	14.01
PYL_MA05	109.01	108.76	16.35	16.15	99.78	96.70	95.85	91.17	81.27	14.58
PYL_MA06	106.30	106.07	15.95	15.75	99.78	96.47	95.94	90.96	80.08	14.21
PYL_MA07	130.93	130.63	19.64	19.40	99.77	96.35	96.12	92.77	87.05	17.51
PYL_MA08	145.47	145.17	21.82	21.56	99.80	96.64	96.27	93.33	88.90	19.46
PYL_MA09	137.75	137.44	20.66	20.42	99.77	96.39	96.29	93.23	88.10	18.43
PYL_MA10	125.77	125.47	18.87	18.64	99.76	96.30	96.16	92.65	86.20	16.82
PYL_MA11	107.23	106.99	16.08	15.88	99.78	96.08	95.83	91.38	81.33	14.34
PYL_MA12	127.11	126.86	19.07	18.85	99.81	96.51	96.31	92.92	86.64	17.01
QT_MS01	127.79	127.40	19.17	18.68	99.69	92.68	94.49	88.27	81.04	16.86
QT_MS02	116.75	116.38	17.51	17.06	99.69	92.42	94.31	87.64	78.81	15.40
QT_MS03	114.56	114.22	17.18	16.79	99.70	92.97	95.17	88.92	79.44	15.15
QT_MS04	115.38	115.08	17.31	16.96	99.74	94.15	95.36	90.25	80.97	15.31
QT_MS05	145.78	145.35	21.87	21.31	99.70	92.72	94.72	89.49	83.97	19.23
QT_MS06	106.84	106.52	16.03	15.69	99.70	93.29	95.77	89.89	78.42	14.16
QT_MS07	123.18	122.84	18.48	18.09	99.72	93.74	95.31	90.11	82.31	16.32
QT_MS08	115.32	114.99	17.30	16.89	99.71	92.71	94.73	88.60	78.80	15.25
QT_MS09	114.63	114.29	17.19	16.77	99.70	92.54	94.43	87.86	78.24	15.14
QT_MS10	124.60	124.23	18.69	18.21	99.71	92.57	94.14	87.90	79.79	16.43
QT_MS11	105.75	105.44	15.86	15.48	99.70	92.41	94.33	87.53	76.13	13.97
QT_MS12	131.85	131.53	19.78	19.37	99.76	94.38	95.69	91.41	84.55	17.49
QY_MH01	114.47	113.93	17.17	16.40	99.53	87.28	90.03	81.08	72.29	14.80
QY_MH02	105.45	104.97	15.82	15.11	99.55	87.29	89.66	80.10	69.66	13.63
QY_MH03	104.64	104.15	15.70	14.99	99.53	87.01	89.70	80.07	69.50	13.53
QY_MH04	127.26	126.69	19.09	18.24	99.55	87.26	90.17	81.89	74.67	16.46

QY_MH05	116.76	116.22	17.51	16.73	99.54	87.30	90.01	81.20	72.77	15.10
QY_MH06	133.93	133.28	20.09	19.20	99.52	86.46	90.83	82.69	76.05	17.33
QY_MH07	115.01	114.49	17.25	16.50	99.54	86.68	90.40	81.27	72.62	14.89
QY_MH08	116.45	115.88	17.47	16.70	99.51	86.11	90.47	81.43	73.07	15.07
QY_MH09	126.13	125.50	18.92	18.08	99.50	86.66	90.61	82.11	74.93	16.32
QY_MH10	125.32	124.74	18.80	17.98	99.54	86.89	90.52	81.88	74.54	16.22
QY_MH11	105.30	104.78	15.80	15.10	99.50	86.84	89.68	80.12	69.77	13.63
QY_MH12	128.81	128.23	19.32	18.49	99.55	86.85	90.30	82.13	75.11	16.69
SG_MS01	141.82	141.32	21.27	20.66	99.65	93.09	94.59	89.20	83.22	18.65
SG_MS02	102.15	101.74	15.32	14.89	99.60	91.45	93.13	85.18	73.26	13.44
SG_MS03	143.05	142.02	21.46	20.74	99.27	92.58	94.37	88.48	82.50	18.72
SG_MS04	168.08	167.47	25.21	24.36	99.63	93.40	93.10	88.24	83.76	21.98
SG_MS05	120.10	119.63	18.01	17.47	99.61	91.77	93.14	86.57	78.46	15.77
SG_MS06	134.66	134.26	20.20	19.67	99.70	93.21	94.14	88.55	81.98	17.75
SG_MS07	127.50	127.10	19.12	18.58	99.69	92.77	92.81	86.51	79.61	16.77
SG_MA08	103.53	103.25	15.53	15.26	99.73	95.48	96.39	90.63	77.22	13.78
SG_MS09	146.05	145.52	21.91	21.28	99.63	92.82	95.03	88.29	82.63	19.21
SG_MS10	113.51	95.77	17.03	13.70	84.37	77.34	92.71	82.46	64.92	12.36
SG_MS11	165.51	160.83	24.83	23.30	97.17	91.26	93.87	88.63	83.59	21.03
SG_MA12	113.87	101.38	17.08	14.80	89.03	83.64	95.79	89.06	72.75	13.36
TEL_MA01	117.43	117.07	17.61	17.41	99.69	96.64	95.99	92.14	84.75	15.71
TEL_MA02	155.78	155.39	23.37	23.10	99.75	96.57	96.44	93.71	90.10	20.85
TEL_MA03	133.28	133.04	19.99	19.78	99.82	96.71	95.96	92.74	87.53	17.86
TEL_MA04	148.44	148.08	22.27	22.02	99.76	96.60	96.35	93.52	89.48	19.87
TEL_MA05	148.19	147.84	22.23	21.98	99.77	96.59	96.51	93.71	89.57	19.84
TEL_MA06	119.87	119.54	17.98	17.77	99.72	96.52	96.09	92.34	85.11	16.04
TEL_MA07	146.81	146.49	22.02	21.77	99.78	96.52	96.32	93.50	89.24	19.65
TEL_MA08	167.03	166.70	25.05	24.77	99.80	96.79	96.08	93.64	90.64	22.36
TEL_MA09	130.69	130.24	19.60	19.35	99.66	96.36	96.33	92.80	87.06	17.47
TEL_MA10	129.37	129.10	19.41	19.14	99.80	95.90	96.40	92.92	86.54	17.28
TEL_MA11	134.93	134.67	20.24	20.02	99.80	96.42	96.34	93.05	87.49	18.06
TEL_MA12	132.71	132.40	19.91	19.68	99.76	96.57	96.10	92.80	87.42	17.76
YD_MS01	114.83	114.45	17.22	16.83	99.68	93.45	95.73	90.15	80.60	15.19
YD_MS02	121.50	121.16	18.23	17.83	99.71	93.60	95.85	91.09	82.61	16.09
YD_MA03	135.42	134.97	20.31	19.79	99.66	92.22	94.85	89.56	83.06	17.86
YD_MA04	133.64	133.27	20.05	19.61	99.72	93.56	95.81	91.33	84.62	17.70
YD_MS05	144.06	143.76	21.61	21.36	99.79	96.57	96.25	93.22	88.76	19.28
YD_MS06	140.65	140.39	21.10	20.85	99.81	96.59	96.06	92.90	88.28	18.82
YD_MA07	166.27	165.76	24.94	24.43	99.69	93.37	96.27	92.81	88.61	22.05
YD_MS08	150.65	150.29	22.60	22.25	99.76	94.81	96.61	93.55	88.72	20.08
YD_MA09	159.45	159.00	23.92	23.43	99.72	93.21	96.06	92.45	87.95	21.15
YD_MS10	184.81	184.42	27.72	27.41	99.79	96.34	96.74	94.57	92.07	24.74
YD_MA11	167.21	166.70	25.08	24.56	99.70	93.08	95.92	92.24	88.20	22.16
YD_MS12	145.68	145.40	21.85	21.60	99.81	96.46	96.22	93.30	88.90	19.50
YNL_MA01	121.83	121.59	18.27	18.08	99.8	96.61	96.13	92.56	85.81	16.32
YNL_MA02	102.09	101.91	15.31	15.16	99.82	96.68	96.15	91.41	79.76	13.68
YNL_MA03	145.30	145.03	21.8	21.57	99.81	96.74	95.98	93.15	89.08	19.47
YNL_MA04	132.74	132.50	19.91	19.70	99.82	96.68	95.98	92.77	87.61	17.78
YNL_MA05	102.66	102.43	15.40	15.23	99.78	96.55	95.63	90.78	79.72	13.75
YNL_MA06	127.89	127.65	19.18	18.98	99.81	96.61	96.07	92.76	87.01	17.13
YNL_MA07	117.72	117.46	17.66	17.45	99.78	96.48	96.03	92.13	84.56	15.75
YNL_MA08	139.78	139.50	20.97	20.75	99.80	96.44	95.94	92.96	88.47	18.73
YNL_MA09	142.34	142.06	21.35	21.12	99.80	96.59	96.35	93.52	89.05	19.06
YNL_MA10	127.85	127.59	19.18	18.98	99.80	96.44	96.12	92.78	86.88	17.13
YNL_MA11	137.01	136.76	20.55	20.33	99.81	96.43	96.14	93.00	88.03	18.35
YNL_MA12	137.99	137.72	20.70	20.47	99.80	96.26	96.05	92.96	88.03	18.48
ZQ_MH01	103.00	101.18	15.45	14.55	98.23	85.36	89.84	79.83	68.59	13.14
ZQ_MH02	129.03	128.41	19.35	18.48	99.52	88.15	90.35	81.93	74.72	16.68
ZQ_MH03	127.06	126.12	19.06	18.15	99.26	87.35	90.19	81.74	74.51	16.38
ZQ_MH04	123.10	122.34	18.47	17.61	99.38	87.39	90.20	81.55	73.81	15.89
ZQ_MH05	129.96	129.00	19.49	18.57	99.26	87.28	90.29	81.99	74.96	16.76

ZQ_MH06	125.11	124.52	18.77	17.92	99.53	88.04	90.52	81.87	74.16	16.17
ZQ_MH07	111.14	110.58	16.67	15.92	99.49	87.26	90.06	80.64	71.18	14.37
ZQ_MH08	136.40	135.77	20.46	19.55	99.54	87.70	90.51	82.51	76.00	17.65
ZQ_MH09	115.61	115.08	17.34	16.58	99.54	87.46	90.77	81.14	72.55	14.96
ZQ_MH10	132.11	131.48	19.82	18.93	99.52	87.69	91.11	82.46	75.40	17.09
ZQ_MH11	112.42	111.88	16.86	16.11	99.52	87.59	90.55	80.85	71.62	14.54
ZQ_MH12	114.17	113.62	17.12	16.37	99.52	87.39	90.94	81.08	72.17	14.77

Table S2. Primers of the described sequences.

Genes	Forward primers	Reversed primers
Aldh3a2	5’ GTGGAGTTGGTAATAGTGGG 3’	5’ GTTTGAGGATGAAAAAGCGT 3’
Acss3	5’ AAAACTTCCTCTGCCTCCCG 3’	5’ TACAAAAAGCCCTCCTCGTC 3’
Hadhb	5’ AAAGTGCCAGGGAAGGATAT 3’	5’ CACCGTCAGTCAGGAAAGAG 3’
Cyp46a1	5’ ACCCATTTTTAGGCTTTTT 3’	5’ CTTCGTCCTCTGCTGTTCTG 3’
Gbe1	5’ CAACAGAAGGAGAGGGGAGG 3’	5’ CCTCCCAGACTGTGAGTGAG 3’
β-actin	5’ ACCCACACCGTGCCCATCTA 3’	5’ CGGACAATTTCTCTTTCGGCTG 3’
Gapdh	5’ GGGAAACTGTGGAGGGATGG 3’	5’ ACCGGTAAGCTTGCCATTGA 3’
18S rRNA	5’ ACACGGAGAGGTAGTGACGA 3’	5’ CCCGAGATCCAACTACGAGC 3’

Table S3. Comparison of stability of reference genes.

Method	Ranking Order (Better--Good--Average)		
	1	2	3
Delta CT	β-actin	Gapdh	18S rRNA
BestKeeper	Gapdh	β-actin	18S rRNA
Normfinder	β-actin	Gapdh	18S rRNA
Genorm	Gapdh | β-actin		18S rRNA
Recommended comprehensive ranking	β-actin	Gapdh	18S rRNA

Table S4. The distribution and number of samples in this study.

Samples	Geographic position	Longitude and latitude	Number
M. amblycephala	Liangzi Lake, Hubei Province (LZL)	114.51, 30.23	12
	Poyang Lake, Jiangxi Province (PYL)	115.80, 28.42	12
	Yuni Lake, Hubei Province (YNL)	112.11, 29.79	12
	Dongting Lake, Hunan Province (DTL)	112.44, 28.82	11
	Tiane Island, Hubei Province (TEL)	112.57, 29.84	12
	Jinsha River Reservoir, Hongan County (JS)	114.62, 31.33	1
	Beijing River, Shaoguan City (SG)	113.59, 24.79	2
	Beijing River, Yingde City (YD)	113.37, 24.06	7
M. skolkovii	Jinsha River Reservoir, Hongan County (JS)	114.62, 31.33	11
	Qiantang River, Zhejiang Province (QT)	120.18, 30.21	12
	Fuyuan, Heilongjiang Province (FY)	134.30, 48.36	12
	Dongting Lake, Hunan Province (DTL)	112.44, 28.82	1
	Beijing River, Shaoguan City (SG)	113.59, 24.79	10
	Beijing River, Yingde City (YD)	113.37, 24.06	5
M. hoffmanni	Xijiang River, Zhaoqing City (ZQ)	112.48, 23.08	12
	Xijiang River, Fengkai City (FK)	111.51, 23.47	12
	Beijing River, Qingyuan City (QY)	113.13, 23.68	12
	Wanquan River, Hainan Island (HN)	110.47, 19.25	12
M. pellegrini	Longxi River, Sichuan Province (LX)	105.49, 28.94	1

Table S5. Data information of genome resequencing.

Sample ID	Raw Reads	Raw bases	Raw GC (%)	Raw Q20(%)	Raw Q30(%)	Clean Reads	Clean bases	Clean GC (%)	Clean Q20(%)	Clean Q30(%)
DTL_MA01	1.36E+08	2.04E+10	38.41	95.32	85.53	1.17E+08	1.76E+10	38.16	97.35	89.44
DTL_MA02	1.49E+08	2.24E+10	38.30	95.86	86.84	1.32E+08	1.98E+10	38.05	97.57	90.18
DTL_MA03	1.72E+08	2.58E+10	38.50	96.32	88.06	1.54E+08	2.31E+10	38.27	97.82	90.98
DTL_MA04	1.52E+08	2.28E+10	38.46	95.82	86.79	1.34E+08	2E+10	38.23	97.57	90.17
DTL_MA05	1.7E+08	2.54E+10	38.37	96.22	87.83	1.52E+08	2.27E+10	38.17	97.78	90.85
DTL_MA06	1.65E+08	2.47E+10	38.30	96.51	88.42	1.48E+08	2.22E+10	38.11	97.94	91.22
DTL_MA07	1.53E+08	2.29E+10	38.75	96.25	87.75	1.37E+08	2.05E+10	38.53	97.79	90.77
DTL_MA08	1.62E+08	2.43E+10	38.46	96.26	87.73	1.45E+08	2.18E+10	38.28	97.79	90.73
DTL_MA09	1.36E+08	2.04E+10	38.51	95.79	86.50	1.19E+08	1.79E+10	38.29	97.58	90.01
DTL_MA10	1.77E+08	2.66E+10	38.38	96.56	88.54	1.6E+08	2.4E+10	38.17	97.93	91.23
DTL_MS11	1.58E+08	2.37E+10	38.31	96.23	87.66	1.41E+08	2.12E+10	38.13	97.79	90.73
DTL_MA12	1.58E+08	2.37E+10	38.21	96.15	87.62	1.39E+08	2.08E+10	37.88	97.78	90.77
FK_MH01	1.52E+08	2.28E+10	38.47	94.84	85.14	1.24E+08	1.86E+10	38.14	97.13	89.32
FK_MH02	1.55E+08	2.32E+10	38.42	94.55	84.40	1.27E+08	1.91E+10	38.04	96.94	88.86
FK_MH03	1.33E+08	2E+10	38.35	94.06	83.30	1.06E+08	1.6E+10	37.97	96.75	88.29
FK_MH04	1.58E+08	2.37E+10	38.32	95.09	85.63	1.33E+08	1.99E+10	37.96	97.17	89.51
FK_MH05	1.49E+08	2.24E+10	38.35	94.74	84.83	1.24E+08	1.85E+10	38.00	97.04	89.14
FK_MH06	1.31E+08	1.96E+10	38.59	93.43	81.91	1.01E+08	1.52E+10	38.23	96.51	87.62
FK_MH07	1.58E+08	2.37E+10	38.61	94.49	84.23	1.3E+08	1.94E+10	38.29	96.91	88.76
FK_MH08	1.49E+08	2.24E+10	38.23	94.73	84.80	1.24E+08	1.86E+10	37.95	97.05	89.17
FK_MH09	1.53E+08	2.3E+10	38.36	94.73	84.77	1.28E+08	1.92E+10	38.05	97.03	89.09
FK_MH10	1.75E+08	2.62E+10	38.73	95.13	85.73	1.47E+08	2.2E+10	38.40	97.21	89.65
FK_MH11	1.39E+08	2.09E+10	38.25	94.06	83.30	1.12E+08	1.68E+10	37.95	96.78	88.38
FK_MH12	1.85E+08	2.77E+10	38.35	95.32	86.20	1.57E+08	2.36E+10	38.07	97.32	89.97
FY_MS01	1.32E+08	1.99E+10	38.36	96.09	87.11	1.17E+08	1.75E+10	38.08	97.73	90.27
FY_MS02	1.41E+08	2.12E+10	38.54	95.46	85.79	1.21E+08	1.82E+10	38.22	97.43	89.58
FY_MS03	1.53E+08	2.29E+10	38.61	95.90	86.96	1.32E+08	1.99E+10	38.33	97.67	90.37
FY_MS04	1.46E+08	2.19E+10	38.49	95.65	86.34	1.26E+08	1.89E+10	38.22	97.56	90.02
FY_MS05	1.57E+08	2.36E+10	38.28	95.99	87.16	1.39E+08	2.08E+10	38.04	97.71	90.49
FY_MS06	1.69E+08	2.54E+10	38.33	96.14	87.56	1.48E+08	2.22E+10	38.05	97.79	90.75
FY_MS07	1.6E+08	2.4E+10	38.30	95.92	86.99	1.41E+08	2.11E+10	38.06	97.67	90.38
FY_MS08	1.67E+08	2.5E+10	38.28	97.34	90.28	1.55E+08	2.32E+10	38.09	98.41	92.47
FY_MS09	1.66E+08	2.49E+10	38.03	97.32	90.17	1.52E+08	2.28E+10	37.76	98.39	92.36
FY_MS10	1.66E+08	2.5E+10	38.16	97.42	90.51	1.54E+08	2.32E+10	37.93	98.43	92.60
FY_MS11	1.6E+08	2.4E+10	38.31	97.12	89.60	1.47E+08	2.21E+10	38.08	98.29	92.01
FY_MS12	1.3E+08	1.95E+10	38.22	96.71	88.41	1.18E+08	1.77E+10	37.96	98.09	91.21
HN_MH01	1.66E+08	2.48E+10	38.30	96.20	87.69	1.46E+08	2.19E+10	38.00	97.79	90.75
HN_MH02	1.62E+08	2.43E+10	38.08	94.57	84.64	1.32E+08	1.98E+10	37.82	97.00	89.12
HN_MH03	1.44E+08	2.16E+10	38.26	94.76	85.06	1.19E+08	1.79E+10	37.97	97.08	89.35
HN_MH04	1.49E+08	2.24E+10	38.24	94.83	85.22	1.24E+08	1.85E+10	37.93	97.08	89.37
HN_MH05	1.57E+08	2.35E+10	38.12	94.56	84.60	1.28E+08	1.93E+10	37.86	97.00	89.12
HN_MH06	1.44E+08	2.16E+10	38.33	94.37	84.18	1.17E+08	1.75E+10	38.05	96.93	88.91
HN_MH07	1.61E+08	2.42E+10	38.22	94.76	85.02	1.34E+08	2.01E+10	37.94	97.04	89.26
HN_MH08	1.5E+08	2.26E+10	38.36	94.68	84.86	1.24E+08	1.86E+10	38.08	97.03	89.24
HN_MH09	1.59E+08	2.39E+10	38.69	94.64	84.78	1.32E+08	1.98E+10	38.43	96.99	89.16
HN_MH10	1.53E+08	2.29E+10	38.71	94.75	85.01	1.27E+08	1.9E+10	38.44	97.05	89.30
HN_MH11	1.24E+08	1.86E+10	38.09	93.82	82.95	1.09E+08	1.63E+10	37.93	95.77	86.28
HN_MH12	1.68E+08	2.52E+10	38.21	95.21	86.09	1.43E+08	2.14E+10	37.95	97.25	89.91
JS_MS01	1.29E+08	1.93E+10	38.51	95.44	85.04	1.11E+08	1.66E+10	38.11	97.31	88.60
JS_MS02	1.6E+08	2.39E+10	38.58	95.34	85.82	1.37E+08	2.05E+10	38.10	97.39	89.70
JS_MS03	1.63E+08	2.44E+10	38.32	95.00	84.97	1.37E+08	2.05E+10	37.85	97.25	89.21
JS_MS04	1.6E+08	2.39E+10	38.50	95.33	85.81	1.37E+08	2.05E+10	38.03	97.41	89.73
JS_MS05	1.45E+08	2.17E+10	38.09	95.29	85.62	1.24E+08	1.86E+10	37.64	97.33	89.48
JS_MS06	1.63E+08	2.45E+10	38.53	95.47	86.15	1.41E+08	2.11E+10	38.05	97.46	89.90
JS_MS07	1.49E+08	2.24E+10	38.30	94.90	84.75	1.25E+08	1.88E+10	37.81	97.20	89.08

JS_MS08	1.67E+08	2.51E+10	38.52	94.83	85.05	1.39E+08	2.09E+10	38.05	97.04	89.14
JS_MA09	2.24E+08	3.36E+10	38.88	95.00	85.85	1.65E+08	2.47E+10	38.37	97.38	90.79
JS_MS10	1.35E+08	2.03E+10	39.01	94.59	84.57	1.11E+08	1.67E+10	38.46	97.01	88.96
JS_MS11	1.43E+08	2.15E+10	38.57	94.76	84.95	1.19E+08	1.79E+10	38.07	97.07	89.14
JS_MS12	1.6E+08	2.4E+10	38.61	96.04	87.27	1.4E+08	2.1E+10	38.34	97.68	90.43
LX_MP01	1.42E+08	2.13E+10	38.31	96.32	87.73	1.27E+08	1.9E+10	38.05	97.93	90.87
LX_MP02	1.36E+08	2.04E+10	38.31	95.40	86.08	1.16E+08	1.74E+10	37.83	97.36	89.71
LX_MP03	1.28E+08	1.92E+10	38.33	95.21	85.57	1.08E+08	1.63E+10	37.91	97.34	89.51
LX_MP04	1.27E+08	1.9E+10	38.46	94.74	84.40	1.05E+08	1.58E+10	38.01	97.10	88.76
LX_MP05	1.16E+08	1.74E+10	38.39	95.17	85.55	1.06E+08	1.59E+10	38.10	96.53	87.84
LX_MP06	1.28E+08	1.91E+10	38.27	94.49	84.32	1.05E+08	1.57E+10	37.75	96.97	88.82
LX_MP07	1.25E+08	1.87E+10	38.51	94.72	84.88	1.04E+08	1.55E+10	37.98	97.07	89.13
LX_MP08	1.4E+08	2.09E+10	38.77	94.61	84.60	1.16E+08	1.74E+10	38.25	96.99	88.93
LX_MP09	1.37E+08	2.05E+10	38.41	94.71	84.80	1.14E+08	1.7E+10	37.90	97.04	89.03
LX_MP10	1.42E+08	2.14E+10	38.78	94.56	84.43	1.17E+08	1.76E+10	38.36	96.98	88.79
LX_MP11	1.46E+08	2.18E+10	38.61	95.05	85.51	1.23E+08	1.84E+10	38.20	97.15	89.34
LX_MP12	1.66E+08	2.49E+10	38.46	95.14	85.77	1.42E+08	2.12E+10	38.11	97.23	89.56
LZL_MA01	1.57E+08	2.35E+10	37.93	95.84	86.83	1.38E+08	2.08E+10	37.65	97.63	90.29
LZL_MA02	1.49E+08	2.23E+10	37.89	95.68	86.45	1.3E+08	1.95E+10	37.62	97.58	90.09
LZL_MA03	1.73E+08	2.6E+10	37.98	96.01	87.33	1.54E+08	2.3E+10	37.71	97.72	90.63
LZL_MA04	1.65E+08	2.47E+10	37.93	96.14	87.62	1.47E+08	2.2E+10	37.71	97.76	90.76
LZL_MA05	1.52E+08	2.28E+10	37.74	95.99	87.22	1.35E+08	2.02E+10	37.51	97.68	90.50
LZL_MA06	1.78E+08	2.67E+10	38.00	96.42	88.32	1.6E+08	2.4E+10	37.76	97.88	91.17
LZL_MA07	1.38E+08	2.07E+10	38.04	95.04	84.83	1.17E+08	1.76E+10	37.75	97.23	89.02
LZL_MA08	1.77E+08	2.65E+10	38.15	96.37	88.16	1.57E+08	2.36E+10	37.88	97.85	91.04
LZL_MA09	1.46E+08	2.19E+10	37.89	95.80	86.74	1.28E+08	1.93E+10	37.66	97.59	90.21
LZL_MA10	1.24E+08	1.86E+10	38.11	95.03	84.25	1.06E+08	1.59E+10	37.82	97.16	88.34
LZL_MA11	1.49E+08	2.24E+10	38.03	95.78	86.19	1.32E+08	1.97E+10	37.79	97.52	89.57
LZL_MA12	1.2E+08	1.8E+10	37.75	95.29	84.92	1.04E+08	1.56E+10	37.50	97.28	88.75
PYL_MA01	1.14E+08	1.71E+10	38.05	95.52	85.23	1.06E+08	1.59E+10	37.83	96.67	87.22
PYL_MA02	1.22E+08	1.83E+10	38.45	94.90	83.74	1.03E+08	1.54E+10	38.08	97.05	87.86
PYL_MA03	1.16E+08	1.74E+10	37.93	95.34	84.78	1E+08	1.51E+10	37.57	97.22	88.40
PYL_MA04	1.14E+08	1.71E+10	38.20	95.17	84.40	1.05E+08	1.57E+10	37.96	96.49	86.68
PYL_MA05	1.26E+08	1.89E+10	38.07	95.32	84.57	1.09E+08	1.64E+10	37.70	97.18	88.13
PYL_MA06	1.24E+08	1.85E+10	38.05	95.26	84.48	1.06E+08	1.59E+10	37.70	97.19	88.14
PYL_MA07	1.53E+08	2.3E+10	38.34	95.22	85.65	1.31E+08	1.96E+10	37.89	97.31	89.51
PYL_MA08	1.69E+08	2.53E+10	38.64	95.58	86.55	1.45E+08	2.18E+10	38.21	97.50	90.13
PYL_MA09	1.61E+08	2.41E+10	38.98	95.41	86.24	1.38E+08	2.07E+10	38.54	97.46	90.00
PYL_MA10	1.46E+08	2.19E+10	38.68	95.51	86.41	1.26E+08	1.89E+10	38.23	97.48	90.05
PYL_MA11	1.19E+08	1.79E+10	38.70	94.65	84.40	1.07E+08	1.61E+10	38.38	96.32	87.20
PYL_MA12	1.49E+08	2.23E+10	38.27	95.38	86.12	1.27E+08	1.91E+10	37.82	97.44	89.92
QT_MS01	1.5E+08	2.25E+10	38.68	95.26	86.01	1.28E+08	1.92E+10	38.30	97.28	89.69
QT_MS02	1.39E+08	2.09E+10	38.50	94.87	85.12	1.17E+08	1.75E+10	38.12	97.10	89.16
QT_MS03	1.4E+08	2.1E+10	38.39	94.48	84.23	1.15E+08	1.72E+10	37.98	96.94	88.69
QT_MS04	1.35E+08	2.03E+10	38.42	95.32	85.90	1.15E+08	1.73E+10	37.96	97.39	89.76
QT_MS05	1.68E+08	2.52E+10	38.21	95.68	86.73	1.46E+08	2.19E+10	37.78	97.51	90.16
QT_MS06	1.19E+08	1.79E+10	38.65	94.39	83.73	1.07E+08	1.6E+10	38.31	96.16	86.73
QT_MS07	1.45E+08	2.17E+10	38.62	95.20	85.64	1.23E+08	1.85E+10	38.16	97.35	89.65
QT_MS08	1.36E+08	2.04E+10	38.93	95.10	85.41	1.15E+08	1.73E+10	38.47	97.32	89.54
QT_MS09	1.35E+08	2.03E+10	39.04	95.15	85.54	1.15E+08	1.72E+10	38.57	97.34	89.61
QT_MS10	1.45E+08	2.18E+10	39.36	95.48	86.37	1.25E+08	1.87E+10	38.84	97.43	89.96
QT_MS11	1.29E+08	1.94E+10	38.57	94.38	83.68	1.06E+08	1.59E+10	38.03	96.98	88.44
QT_MS12	1.53E+08	2.3E+10	38.37	95.56	86.48	1.32E+08	1.98E+10	37.91	97.47	90.01
QY_MH01	1.4E+08	2.1E+10	38.34	94.42	84.13	1.14E+08	1.72E+10	37.87	96.85	88.55
QY_MH02	1.32E+08	1.99E+10	38.36	93.94	83.09	1.05E+08	1.58E+10	37.84	96.65	88.02
QY_MH03	1.3E+08	1.95E+10	38.11	94.10	83.38	1.05E+08	1.57E+10	37.64	96.69	88.11
QY_MH04	1.56E+08	2.34E+10	38.29	94.31	83.92	1.27E+08	1.91E+10	37.82	96.81	88.49
QY_MH05	1.43E+08	2.15E+10	38.54	94.30	83.82	1.17E+08	1.75E+10	38.03	96.76	88.33
QY_MH06	1.57E+08	2.36E+10	38.30	95.32	86.00	1.34E+08	2.01E+10	37.90	97.38	89.80
QY_MH07	1.37E+08	2.06E+10	38.40	94.95	85.08	1.15E+08	1.73E+10	37.97	97.21	89.25
QY_MH08	1.39E+08	2.09E+10	38.41	94.88	85.00	1.16E+08	1.75E+10	37.99	97.21	89.27
QY_MH09	1.47E+08	2.2E+10	38.20	95.49	86.39	1.26E+08	1.89E+10	37.82	97.45	90.00

QY_MH10	1.45E+08	2.18E+10	38.36	95.47	86.32	1.25E+08	1.88E+10	37.97	97.42	89.92
QY_MH11	1.27E+08	1.91E+10	38.30	94.63	84.27	1.05E+08	1.58E+10	37.81	97.06	88.73
QY_MH12	1.46E+08	2.19E+10	38.24	95.93	86.97	1.29E+08	1.93E+10	38.02	97.65	90.27
SG_MS01	1.73E+08	2.6E+10	38.64	95.64	86.74	1.42E+08	2.13E+10	37.96	97.50	90.13
SG_MS02	1.23E+08	1.85E+10	38.59	94.76	84.54	1.02E+08	1.53E+10	38.04	97.12	88.88
SG_MS03	1.69E+08	2.53E+10	38.55	96.18	87.42	1.43E+08	2.15E+10	38.10	97.75	90.39
SG_MS04	2.06E+08	3.09E+10	38.91	96.95	89.53	1.68E+08	2.52E+10	38.21	98.14	91.75
SG_MS05	1.4E+08	2.11E+10	38.34	95.83	86.39	1.2E+08	1.8E+10	37.96	97.52	89.62
SG_MS06	1.54E+08	2.32E+10	38.33	96.27	87.65	1.35E+08	2.02E+10	37.96	97.82	90.63
SG_MS07	1.45E+08	2.18E+10	38.29	96.21	87.47	1.27E+08	1.91E+10	37.98	97.79	90.54
SG_MA08	1.23E+08	1.84E+10	38.39	95.33	85.19	1.04E+08	1.55E+10	38.01	97.36	89.10
SG_MS09	1.67E+08	2.5E+10	38.49	96.42	88.07	1.46E+08	2.19E+10	38.11	97.91	90.93
SG_MS10	1.32E+08	1.98E+10	37.86	95.60	87.17	1.14E+08	1.7E+10	37.39	96.99	89.68
SG_MS11	2.05E+08	3.08E+10	38.64	96.90	89.54	1.66E+08	2.48E+10	37.92	98.16	91.84
SG_MA12	1.35E+08	2.02E+10	37.96	95.37	86.71	1.14E+08	1.71E+10	37.42	96.93	89.52
TEL_MA01	1.35E+08	2.02E+10	38.02	95.56	86.04	1.17E+08	1.76E+10	37.82	97.45	89.70
TEL_MA02	1.74E+08	2.62E+10	38.22	96.24	87.80	1.56E+08	2.34E+10	38.03	97.79	90.82
TEL_MA03	1.51E+08	2.26E+10	38.40	96.03	87.26	1.33E+08	2E+10	38.19	97.69	90.47
TEL_MA04	1.66E+08	2.49E+10	38.53	96.28	87.93	1.48E+08	2.23E+10	38.32	97.81	90.90
TEL_MA05	1.67E+08	2.51E+10	38.42	96.13	87.54	1.48E+08	2.22E+10	38.20	97.73	90.63
TEL_MA06	1.39E+08	2.08E+10	38.49	95.53	85.96	1.2E+08	1.8E+10	38.25	97.45	89.67
TEL_MA07	1.65E+08	2.47E+10	38.54	96.34	88.04	1.47E+08	2.2E+10	38.29	97.83	90.92
TEL_MA08	1.87E+08	2.8E+10	38.54	96.53	88.56	1.67E+08	2.51E+10	38.27	97.92	91.25
TEL_MA09	1.5E+08	2.25E+10	38.45	95.71	86.41	1.31E+08	1.96E+10	38.18	97.56	89.98
TEL_MA10	1.46E+08	2.19E+10	38.27	96.16	87.57	1.29E+08	1.94E+10	38.04	97.75	90.65
TEL_MA11	1.53E+08	2.29E+10	38.84	95.99	87.27	1.35E+08	2.02E+10	38.54	97.70	90.57
TEL_MA12	1.53E+08	2.3E+10	38.52	95.70	86.48	1.33E+08	1.99E+10	38.19	97.57	90.08
YD_MS01	1.4E+08	2.1E+10	38.23	94.44	84.13	1.15E+08	1.72E+10	37.95	96.93	88.81
YD_MS02	1.46E+08	2.18E+10	38.09	94.72	84.83	1.22E+08	1.82E+10	37.84	97.03	89.16
YD_MA03	1.62E+08	2.43E+10	38.30	94.84	85.12	1.35E+08	2.03E+10	38.05	97.09	89.34
YD_MA04	1.57E+08	2.36E+10	37.91	95.14	85.77	1.34E+08	2E+10	37.70	97.20	89.65
YD_MS05	1.7E+08	2.55E+10	38.47	95.06	85.63	1.44E+08	2.16E+10	38.21	97.17	89.58
YD_MS06	1.66E+08	2.49E+10	38.44	95.18	85.92	1.41E+08	2.11E+10	38.18	97.25	89.80
YD_MA07	1.82E+08	2.73E+10	38.32	96.87	89.25	1.66E+08	2.49E+10	38.09	98.16	91.79
YD_MS08	1.65E+08	2.48E+10	38.42	96.79	89.01	1.51E+08	2.26E+10	38.20	98.14	91.66
YD_MA09	1.72E+08	2.59E+10	38.15	97.17	90.03	1.59E+08	2.39E+10	37.93	98.31	92.32
YD_MS10	1.98E+08	2.98E+10	38.26	97.41	90.80	1.85E+08	2.77E+10	38.05	98.44	92.84
YD_MA11	1.8E+08	2.7E+10	38.05	97.24	90.25	1.67E+08	2.51E+10	37.84	98.34	92.46
YD_MS12	1.63E+08	2.45E+10	38.69	96.22	87.80	1.46E+08	2.19E+10	38.46	97.76	90.80
YNL_MA01	1.44E+08	2.16E+10	38.24	95.13	85.49	1.22E+08	1.83E+10	37.81	97.33	89.55
YNL_MA02	1.21E+08	1.81E+10	38.54	95.06	85.22	1.02E+08	1.53E+10	38.14	97.28	89.34
YNL_MA03	1.66E+08	2.5E+10	38.26	95.78	87.01	1.45E+08	2.18E+10	37.90	97.62	90.43
YNL_MA04	1.54E+08	2.32E+10	38.35	95.39	86.06	1.33E+08	1.99E+10	37.97	97.43	89.85
YNL_MA05	1.17E+08	1.75E+10	38.02	95.89	86.32	1.03E+08	1.54E+10	37.83	97.61	89.68
YNL_MA06	1.49E+08	2.23E+10	38.40	95.46	86.24	1.28E+08	1.92E+10	38.02	97.48	89.99
YNL_MA07	1.4E+08	2.09E+10	38.71	95.25	85.73	1.18E+08	1.77E+10	38.26	97.40	89.69
YNL_MA08	1.58E+08	2.37E+10	38.22	96.10	87.47	1.4E+08	2.1E+10	37.98	97.76	90.66
YNL_MA09	1.6E+08	2.4E+10	38.39	96.23	87.77	1.42E+08	2.14E+10	38.16	97.82	90.83
YNL_MA10	1.45E+08	2.18E+10	38.41	95.82	86.72	1.28E+08	1.92E+10	38.18	97.62	90.20
YNL_MA11	1.54E+08	2.31E+10	38.33	96.11	87.44	1.37E+08	2.06E+10	38.09	97.75	90.61
YNL_MA12	1.56E+08	2.34E+10	38.57	96.04	87.30	1.38E+08	2.07E+10	38.33	97.74	90.58
ZQ_MH01	1.32E+08	1.98E+10	38.18	93.67	82.60	1.03E+08	1.54E+10	37.94	96.62	88.04
ZQ_MH02	1.59E+08	2.38E+10	38.55	94.69	84.85	1.29E+08	1.94E+10	38.21	96.98	89.08
ZQ_MH03	1.54E+08	2.31E+10	38.12	94.72	84.94	1.27E+08	1.91E+10	37.85	97.01	89.19
ZQ_MH04	1.51E+08	2.26E+10	38.46	94.63	84.69	1.23E+08	1.85E+10	38.10	96.94	88.96
ZQ_MH05	1.57E+08	2.35E+10	38.38	94.81	85.12	1.3E+08	1.95E+10	38.09	97.04	89.27
ZQ_MH06	1.54E+08	2.31E+10	38.54	94.65	84.79	1.25E+08	1.88E+10	38.18	96.97	89.07
ZQ_MH07	1.38E+08	2.07E+10	38.48	94.33	84.07	1.11E+08	1.67E+10	38.20	96.87	88.75
ZQ_MH08	1.65E+08	2.47E+10	38.39	94.95	85.42	1.36E+08	2.05E+10	38.11	97.20	89.55
ZQ_MH09	1.4E+08	2.1E+10	38.20	94.70	84.82	1.16E+08	1.73E+10	37.97	97.07	89.18
ZQ_MH10	1.59E+08	2.39E+10	38.60	94.92	85.34	1.32E+08	1.98E+10	38.31	97.15	89.44
ZQ_MH11	1.37E+08	2.05E+10	38.44	94.79	85.03	1.12E+08	1.69E+10	38.15	97.10	89.27

ZQ_MH12	1.41E+08	2.12E+10	38.43	94.42	84.17	1.14E+08	1.71E+10	38.13	96.93	88.75

Table S6. Variation information of samples in this study.

		Total	*M. amblycephala*	*M. hoffmanni*	*M. pellegrini*	*M. skolkovii*
**accession number**		180	69	48	12	51
SNP	SUM	31,857,189	16,235,392	16,133,317	6,825,670	17,179,046
	Intergenic	17,662,510	9,096,237	8,883,853	3,865,100	9,717,098
	Intronic	13,067,222	6,531,879	6,625,127	2,700,354	6,837,535
	3'UTR	89,504	43,219	42,060	15,415	42,908
	5'UTR	49,587	23,780	23,851	8,967	23,853
	Nonsynonymous	452,201	258,010	271,477	119,976	273,031
	synonymous	481,078	254,026	258,221	104,844	256,836

Table S7. Candidate genes and enriched pathways in selective sweep analysis.

**Genes**	**PiR value**	**XP-EHH value**	**Pathways**
Fatty aldehyde dehydrogenase (*Aldh3a2*)	2.57	-1.91	Valine, leucine and isoleucine degradation; Glycolysis/gluconeogenesis; Phytol degradation
Long chain fatty acid-CoA ligase (*Acsbg2*)	1.72	-1.64	Fatty acid biosynthesis
Protein kinase B (*Akt2*)	1.46	-1.71	Insulin signaling pathway
Glycogen branching enzyme (*Gbe1*)	2.30	-2.38	Starch and sucrose metabolism
Mitochondrial tri-functional protein β-subunit (*Hadhb*)	-2.11	1.18	Glycolysis/gluconeogenesis; Fatty acid degradation
Acyl-CoA synthetase short-chain 3 (*Acss3*)	1.89	-2.01	Phytol degradation
Cholesterol 24-hydroxylase (*Cyp46a1*)	1.48	-2.10	Cholesterol metabolism
Bile acid CoA: amino acid N-acyltransferase (*Baat*)	1.12	-2.09	Cholesterol metabolism
Umami Taste Receptor gene (*Tas1r1*)	0.30	-1.32	Sensory system
